# *TICRR* Contributes to Tumorigenesis Through Accelerating DNA Replication in Cancers

**DOI:** 10.3389/fonc.2019.00516

**Published:** 2019-06-18

**Authors:** Qin Yu, Shao-Yan Pu, Huan Wu, Xiao-Qiong Chen, Jian-Jun Jiang, Kang-Shuyun Gu, Yong-Han He, Qing-Peng Kong

**Affiliations:** ^1^State Key Laboratory of Genetic Resources and Evolution/Key Laboratory of Healthy Aging Research of Yunnan Province, Kunming Institute of Zoology, The Chinese Academy of Sciences, Kunming, China; ^2^Center for Excellence in Animal Evolution and Genetics, Chinese Academy of Sciences, Kunming, China; ^3^Kunming Key Laboratory of Healthy Aging Study, Kunming, China; ^4^KIZ/CUHK Joint Laboratory of Bioresources and Molecular Research in Common Diseases, Kunming, China; ^5^Kunming College of Life Science, University of Chinese Academy of Sciences, Beijing, China

**Keywords:** *TICRR*, DNA replication, proliferation, p53 pathway, tumorigenesis, ATM/CHK2

## Abstract

DNA replication is precisely regulated in cells and its dysregulation can trigger tumorigenesis. Here we identified that the TOPBP1 interacting checkpoint and replication regulator (*TICRR*) mRNA level was universally and highly expressed in 15 solid cancer types. Depletion of *TICRR* significantly inhibited tumor cell growth, colony formation and migration *in vitro*, and strikingly inhibited tumor growth in the xenograft model. We reveal that knockdown of *TICRR* inhibited not only the initiation but also the fork progression of DNA replication. Suppression of DNA synthesis by *TICRR* silencing caused DNA damage accumulation, subsequently activated the ATM/CHK2 dependent p53 signaling, and finally induced cell cycle arrest and apoptosis at least in p53-wild cancer cells. Further, we show that a higher *TICRR* level was associated with poorer overall survival (OS) and disease free survival (DFS) in multiple cancer types. In conclusion, our study shows that *TICRR* is involved in tumorigenesis by regulating DNA replication, acting as a common biomarker for cancer prognosis and could be a promising target for drug-development and cancer treatment.

## Introduction

Rapid proliferation of cancer cells requires DNA hyper-replication, which may lead to genomic instability and promote tumor development (1). Among the three interdependent and sequential events of DNA replication, i.e., licensing, firing and progression (2), initiation phase (including licensing and firing) is the rate-limiting step (3). Accordingly, the initiation regulators likely play a crucial role in tumorigenesis by modulating the origin-firing timing during DNA replication in cancer cells (4).

Indeed, previous studies have revealed that high expression of initiation factors could promote dormant origins fire early, which can shorten replication timing and accelerate cell proliferation (4–6). Among the identified replication initiation factors, TICRR (also known as Treslin in vertebrate and sld3 in yeast) is likely a hub one (7), as this protein mediates not only the assembly of CMG (CDC45-MCM2-7-GINS) helicase complex by recruiting CDC45 and GINS (3, 8–10), but also the activation of the complex via stimulating MCM2 phosphorylation (11). Recent studies revealed that TICRR/Treslin determined S-phase progression from expression level to epigenetic control (12–14). Given its key role in DNA replication, we hypothesize that over-expression of *TICRR* may contribute to rapid cellular proliferation of cancer cells via accelerating the hyper-replication of DNA. Indeed, among the numerous differentially expressed genes (DEGs) recently identified via analyzing 5,540 cancerous transcriptomes (15), we found that *TICRR* is consistently up-regulated expression across all the cancer types under analysis.

To gain insights into the mechanism of *TICRR* in tumorigenesis, we manipulated *TICRR* gene expression and found that *TICRR*-depletion cells displayed strikingly reduced cell proliferation *in vitro* and tumorigenic growth *in vivo*, via suppressing DNA replication and activating ATM/CHK2 dependent p53 pathway. In addition, higher expression of *TICRR* predicts poor clinical outcome, making it a promising marker for cancer prognosis.

## Materials and Methods

### Cell Culture

The breast cancer cell line MCF7 was cultured in DMEM/high glucose (SH30243.01B, HyClone, Logan, UT) with 10% fetal bovine serum (FBS) (04-001-1ACS, Biological Industries, Kibbutz Beth Haemek, Israel) and 1% penicillin/streptomycin (p/s) (C0222, Beyotime Institute of Biotechnology, Jiangsu, China). SKBR3, HCC1806 and 786-0 cells were cultured in RPMI-1640 (SH30809.01B, HyClone) containing 10% FBS and 1% p/s. MDA-MB-231 cells were cultured in DMEM/F12 (SH30023.01B, HyClone), supplemented with 10% FBS and 1% p/s. The immortalized human breast epithelial cell line MCF10A was maintained in DMEM/F12, supplemented with 5% horse serum (16050-130, Gibco, New Zealand), 20 ng/mL EGF (PHG0311, Invitrogen, Carlsbad, CA), 0.5 mg/mL hydrocortisone (MB1567, Meilunbio, Dalian, China), 100 ng/mL cholera toxin (C8052, Sigma-Aldrich, St. Louis, MO), 10 μg/mL insulin (Wanbang Biopharmaceuticals, Xuzhou, China), and 1% p/s. The cells were purchased from Conservation Genetics CAS Kunming Cell Bank. Cell lines were tested to be mycoplasma-free by PCR (16).

### RNA Interference

For siRNA experiments, cells were transfected with two *TICRR*-specific siRNA and control siRNA (RiboBio, Guangzhou, China) at a final concentration of 50 nM using ribo*FECT*™ CP Transfection Kit (C10511-1, RiboBio) according to the manufacturer's instructions. The sequences of the siRNA were listed in Table S1. For generation of stable cell population, the shRNA targeting *TICRR* was cloned into pLKO.1 lentiviral vector. The lentiviruses were generated from HEK-293T cells and collected at 48 h and 72 h after transfection. Then cells were infected with lentiviruses, and selected in the presence of puromycin for three generations.

### Quantitative RT-PCR (RT-qPCR)

Total RNA were extracted using TRIzol reagent (11667165001, Invitrogen), followed by treatment of DNase I (EN0521, Thermo scientific, MA, USA). Reverse transcription was performed with oligo (dT) primers using GoScript^TM^ reverse transcription system (A5001, Promega, Madison, WI) according to the manufacturer's protocol. Quantitative real-time PCR with gene-specific primers was performed using GoTaq® qPCR Master Mix (A6002, Promega). The comparative C_T_ method was applied for quantification of gene expression, and values were normalized to beta actin (*ACTB*). The primers used in the study were shown in Table S2.

### Immunoblotting

Cells were lysed with RIPA Lysis Buffer with PMSF (ST506, Beyotime Institute of Biotechnology). Proteins were quantified by BCA Protein Assay Kit (P0010, Beyotime Institute of Biotechnology). 20–80 μg of total protein was loaded onto SDS-PAGE and subsequently transferred to PVDF membranes (162-0177, Bio-Rad, Richmond, CA). After blocking with 5% nonfat milk or bovine serum albumin (BSA) in PBST, membranes were incubated with the following primary antibodies at the suggested dilutions: anti-CDKN1A/p21 (A1483, ABclonal, Cambridge, MA), anti-TP53/p53 (sc-6243, Santa Cruz Biotechnology, Santa Cruz, CA), anti-ERK1/2 (A0229, ABclonal), anti-p-ERK1/2 (AP0472, ABclonal), anti-p-Histone H2A.X (S139) (AP0099, ABclonal), anti-p-ATM (10H11.E12) (sc-47739, Santa Cruz Biotechnology), anti-p-CHK1 (Ser345) (sc-17922, Santa Cruz Biotechnology), anti-p-CHK2 (Thr68) (sc-16297-R, Santa Cruz Biotechnology), anti-PUMA (sc-374223, Santa Cruz Biotechnology), anti-TICRR (NBP2-41283, Novus Biologicals, Littleton, CO), anti-cyclin D1 (2922S, Cell Signaling Technology, Danver, MA) and anti-ACTB (AA128, Beyotime Institute of Biotechnology). Primary antibodies were detected with HRP-labeled goat anti-rabbit (A0208, Beyotime Institute of Biotechnology) or anti-mouse IgG (H+L) (A0216, Beyotime Institute of Biotechnology).

### Cell Viability Assay and DNA Synthesis

Cells were seeded in 96-well plates at a density of 3 × 10^3^ cells. Then, cell viability were quantified by CellTiter 96® AQueous One Solution Cell Proliferation Assay (G3580, Promega) with 490 nm plate reading at indicated time points according to the manufacturer's protocol. Cells were seeded into 96-well plates and DNA synthesis was measured using Cell-Light™ EdU Apollo®488 *in vitro* Imaging Kit (100T) (C10310-3, RiboBio) following the manufacturer's protocols. In brief, cells were labeled with 50 μM EdU for 2 h, then fixed in 4% paraformaldehyde for 30 min, then stained with Apollo®488 and Hoechst 33342. Cells were imaged by Nikon eclipse Ti inverted microscope.

### Cell Cycle and Apoptosis Assay

For cell cycle analysis, cells were collected after transfection, then washed and fixed with cold 75% alcohol overnight. After wash with PBS, cells were labeled with propidium iodide (PI) (P4170-10, Sigma-Aldrich) and incubated at room temperature in the dark for 30 min. Cells were then filtered through a nylon mesh filter and subjected to flow cytometry (BD Biosciences). For cell apoptosis analysis, cells were harvested at 48 h after transfection, and stained using the FITC-Annexin V apoptosis detection kit and PI staining solution (88-8005-72, eBioscience, San Diego, CA) according to manufacturer's protocol. FACS (fluorescence activated cell sorter) analysis was performed within 4 h and the results were analyzed by FlowJo software (Version 7.6.1).

### DNA Fiber Assay

MCF7 cells were transfected with *TICRR*-specific siRNA and control siRNA for 48 h, then labeled with 50 μM IdU (I7125, Sigma-Aldrich) for 20 min. DNA fiber spreads were performed as previously reported (17, 18). Briefly, cell were harvested and re-suspended with cold PBS. 2.5 μL of the cell suspension was spotted onto a glass slide and mixed with 7.5 μL lysis solution (0.5% SDS, 50 mM EDTA, 200 mM Tris-HCl). Slides were then tilted to 15° to allow the fibers to spread. Fibers were air-dried and fixed in methanol and acetic acid (3:1) and subsequently acid treated with hydrochloric acid (2.5 N) to denature the DNA fibers. Later, slides were stained with immunofluorescent anti-BrdU (347580, BD Biosciences, San Jose, CA). Slides were imaged at 60 × using an Olympus FluoView™ FV1000. Fiber length was analyzed using Image-Pro Plus software (Version 6.0). Table S3 shows the number of fibers and independent experiments performed under each condition. The median and mean of replication tract length and *p*-values derived from the Welch's two-tailed *t*-test were calculated using R software.

### Colony Formation Assay

For colony formation assay, cells after transfection were seeded at 500 cells per well in a 6-well plate and incubated for 15 d. Cells were fixed with fixative (methyl alcohol: glacial acetic acid = 3:1) for 15 min and then stained with 0.1% crystal violet for 20 min. The colony formation rate was calculated as colony number/cell number seeded.

### Migration Assays

Cell migration was evaluated by wound healing and transwell assays. For the wound healing assay, transfected cells were seeded in 6-well plates and cultured to 90% confluence. Cells were scraped with a 200 μL tip and washed with PBS for 3 times, and then cultured in fresh medium containing 2% serum for 24 h. Photographs were took at 0 h and 24 h. The width was measured with Image-Pro Plus software. The relative migration in *TICRR* knockdown cells was normalized to the control cells. For transwell migration assay, 24-well polycarbonate inserts were used. After transfection, cells were cultured on the top chamber of 24-well transwell plate (3422, Corning, Glendale, AZ) in 2% FBS medium and medium with 20% FBS was added into the bottom chambers. After 24 h, the cells on the surface of top chamber membrane were removed with a cotton swab. The migrated cells on the bottom surface of chamber membrane were fixed with 4% paraformaldehyde for 20 min, stained with 0.1% crystal violet for 20 min, washed with PBS and air dried. The crystal violet was dissolved with 500 μL 33% acetic acid, and the OD570 nm value was recorded.

### Tumorigenesis Assay

Tumor xenografts were performed by injecting shControl-HCC1806 cells and shTICRR-HCC1806 cells (1.5 × 10^6^ cells per 100 μL DMEM with 30% BD matrigel) into subcutaneous of 5-week-old female BALB/c nude mice (Vital River, Beijing, China). Once tumors were detectable, the mice were monitored and the tumor volumes (V) were measured twice a week by determining length (L) and width (W) using Vernier calipers and calculated using the formula: V = L × W^2^/2. One month later, mice were sacrificed, and the tumors were excised for mass measurement and imaging. The mouse experiment was approved by the animal ethics committee of the Kunming Institute of Zoology, Chinese Academy of Sciences.

### Data Acquisition

Gene expression data and clinical data of 15 cancer types were downloaded from The Cancer Genome Atlas (TCGA, https://tcga.xenahubs.net). Overall survival (OS) analysis was performed in 15 cancer types using a web-tool OncoLnc (http://www.oncolnc.org) (19), and the disease free survival (DFS) was analyzed using R software. The samples were grouped by the median of *TICRR* expression. For survival analyses, log-rank tests were used to determine the statistical significance.

### RNA Sequencing (RNA-seq)

Total RNA was isolated using TRIzol reagent and RNA sequencing was completed by Novogene Company. The process of data analysis was described previously (20).

### Statistics

Data were expressed as means ± SEM. Statistical analyses were performed using GraphPad Prism 7.0 (GraphPad Software) or R software (version 3.3.2). *P* < 0.05 were considered statistically significant.

## Results

### *TICRR* Is Universally and Highly Expressed in Various Tumors

**Figure 1:**
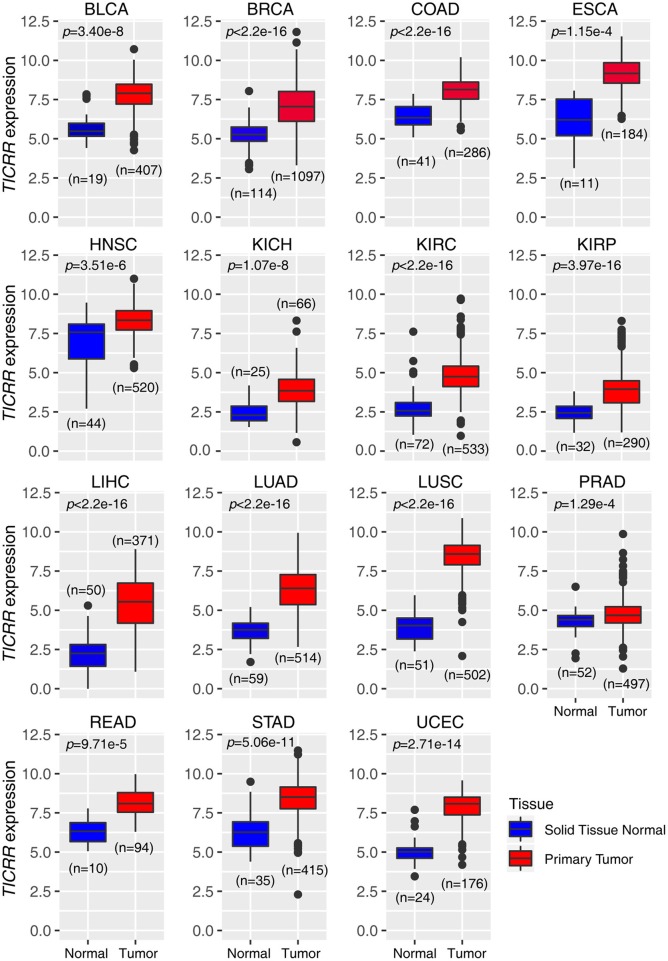
Expression patterns of *TICRR* across 15 tumor types. The expression of *TICRR* was shown as log2(norm_count + 1). *P*-values were determined as two-tailed Student's *t*-test. Data from The Cancer Genome Atlas were downloaded at https://tcga.xenahubs.net. BLCA, bladder urothelial carcinoma; BRCA, breast invasive carcinoma; COAD, colon adenocarcinoma; ESCA, Esophageal carcinoma; HNSC, head and neck squamous cell carcinoma; KICH, kidney chromophobe; KIRC, kidney renal clear cell carcinoma; KIRP, kidney renal papillary cell carcinoma; LIHC, liver hepatocellular carcinoma; LUAD, lung adenocarcinoma; LUSC, lung squamous cell carcinoma; PRAD, prostate adenocarcinoma; READ, Rectum adenocarcinoma; STAD, Stomach adenocarcinoma; UCEC: uterine corpus endometrial carcinoma.

In agreement with our previous observation based on a newly developed algorithm (15), re-analysis of the *TICRR* expression data extracted from TCGA database did show that this gene mRNA level was strikingly up-regulated in all of the 15 cancer types compared to the normal tissues (all *p* < 1.00 × 10^−3^) (Figure 1). Based on the expression profile, we tested *TICRR* expression using quantitative real-time PCR assay (RT-qPCR) in several cell lines, including the immortalized human breast epithelial cell line (MCF10A), four breast cancer cell lines (MCF7, SKBR3, HCC1806, and MDA-MB-231), and one kidney cancer cell line (786-0). The results showed that *TICRR* displayed the highest expression in MCF7 cells (*p* = 3.30 × 10^−3^, compared to MCF10A), followed by HCC1806 (*p* = 0.017) (Figure S1A). At the protein level, TICRR expression in MCF7 cells was also higher than that in MCF10A cells (Figure S1B).

### *TICRR* Knockdown Inhibits Cancer Cell Viability *in vitro*

**Figure 2:**
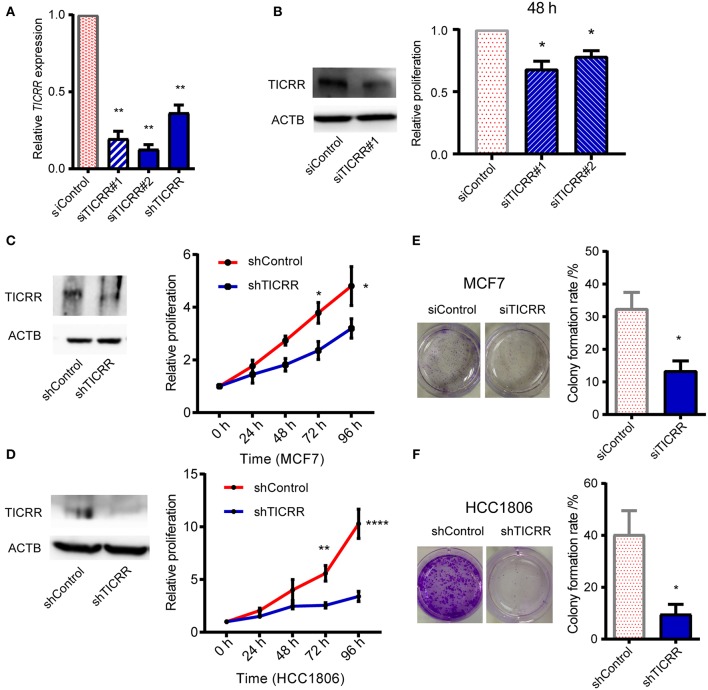
Knockdown of *TICRR* inhibited cell proliferation. **(A)** *TICRR* expression was inhibited in MCF7 cells using siRNA and shRNA. Efficiency of *TICRR* knockdown evaluated by RT-qPCR. Data were mean ± SEM, *N* = 3 biological replicates. **(B)** Relative proliferation in control and *TICRR*-knockdown MCF7 cells after transfection for 48 h. Western blot (left) was performed. The proliferation in control cells was set at 1. *P < 0.05, two-tailed Student's *t*-test. Data were mean ± SEM, *N* = 3 biological replicates. **(C,D)** Growth curves of *TICRR*-knockdown cells and control in MCF7 **(C)** and HCC1806 **(D)** cell lines. western blot (left) was performed. Cell viability at 0 h was set at 1. *P < 0.05, **p < 0.01, ****p < 1.0 × 10^−4^, two-way ANOVA test. Results were displayed as means ± SEM, *N* = 3 biological replicates. **(E,F)** Knockdown of *TICRR* inhibited the colony formation in MCF7 **(E)** and HCC1806 **(F)** cells examined by plate colony formation assay. Representative colonies pictures were shown on the left and percentage of colony number were on the right. *P < 0.05, two-tailed Student's *t*-test. Results were displayed as means ± SEM, *N* = 3 biological replicates.

To investigate the role of *TICRR* in cancer cell growth, shRNA and siRNA mediated *TICRR* knockdown were performed in MCF7 cells (Figure 2A). Knock down efficiency was validated in other cell lines (Figure S2A). *TICRR* silencing caused 20–30% inhibition of proliferation in MCF7 cells compared to the control cells at 48 h after transfection with siRNA (all *P* < 0.05) (Figure 2B). Cell growth curves of MCF7 and HCC1806 (Figures 2C,D), SKBR3, MCF10A, and 786-0 cells (Figures S2B,C) consistently showed the inhibitory effect of *TICRR* knockdown on cell growth, with 30~65% inhibition at 96 h (all *P* < 0.05). We further examined cell apoptosis to test whether the reduced cell viability by *TICRR* knockdown is attributed to cell death. Results showed that the percentage of apoptotic cells was significantly increased in *TICRR*-knockdown cells (*p* = 8.70 × 10^−3^, Figure S3), suggesting enhancement of cell death through apoptosis. We further examined the ability of *TICRR* to regulate colony formation. Foci number in *TICRR*-knockdown cells was significantly reduced in MCF7 (*p* = 0.02) (Figure 2E), which was validated in HCC1806 cells (*p* = 0.04) (Figure 2F). Furthermore, we found that *TICRR* depletion significantly inhibited cancer cell migration *in vitro* as revealed by the wound healing (Figure S4A) and transwell assays (Figure S4B).

### *TICRR* Knockdown Significantly Inhibits Tumorigenesis *in vivo*

**Figure 3:**
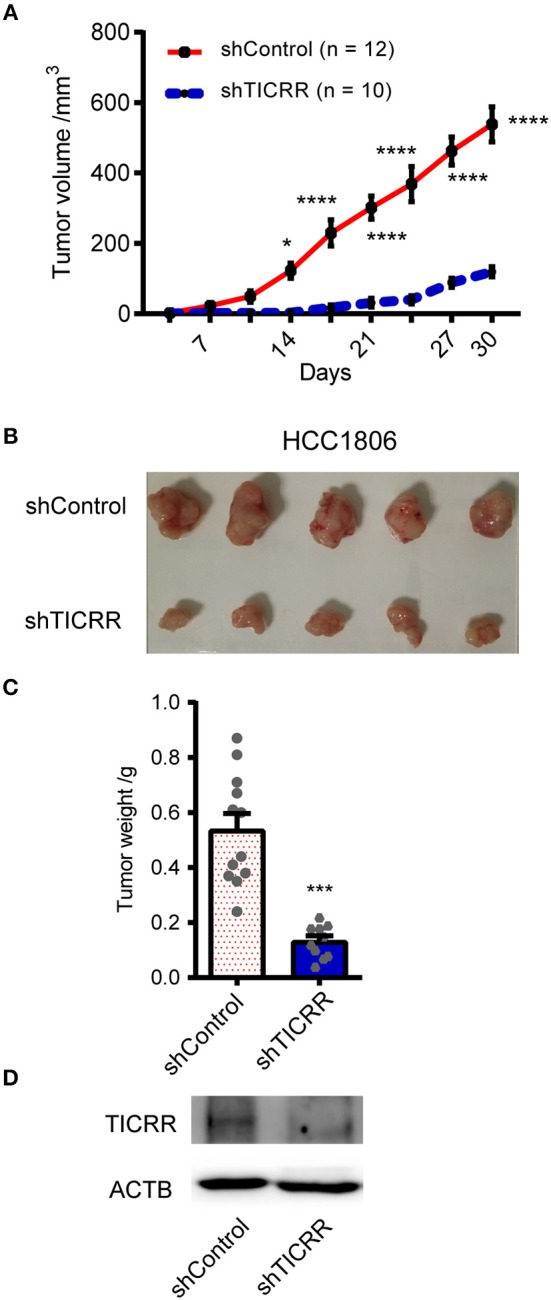
Knockdown of *TICRR* inhibited tumor growth *in vivo*. **(A)** The xenograft tumor volume vs. days of post-injection was shown from BALB/c nude mice injected with shTICRR- and shControl- HCC1806 cells. *p-value < 0.05, ****p-value < 1.00 × 10^−4^, two-way ANOVA test. Results were displayed as means ± SEM. **(B)** Representative photo of xenograft tumors. **(C)** Tumor weight. The tumor-bearing mice were sacrificed and the tumors were dissected out at the last day. The number of mice is 12 and 10 for the shControl and shTICRR group, respectively. ***p-value < 1.00 × 10^−4^, two-tailed Student's *t* test. Results were displayed as means ± SEM. **(D)** TICRR level in tumors harvested from mice at the last day.

To explore the role of *TICRR* in affecting tumor growth *in vivo*, we injected shTICRR- and shControl-HCC1806 cells into BALB/c nude mice and found that the growth of xenograft tumors was strikingly slower in mice injected with shTICRR-HCC1806 cells (tumor volume on the last day: control = 538.4 ± 49.68 mm^3^, *TICRR*-depletion = 119.4 ± 14.56 mm^3^, *p* = 2.12 × 10^−6^) (Figures 3A,B). Consistently, the tumor weight in the shTICRR group (0.13 ± 0.02 g) was dramatically reduced than that in the control (0.54 ± 0.06 g) (*p* = 1.43 × 10^−5^) (Figure 3C). The TICRR protein was still lower in the shTICRR tumors compared to the control (Figure 3D). These results collectively support a critical role of *TICRR* in tumorigenesis *in vivo*.

### Knockdown of *TICRR* Significantly Inhibits DNA Replication

**Figure 4:**
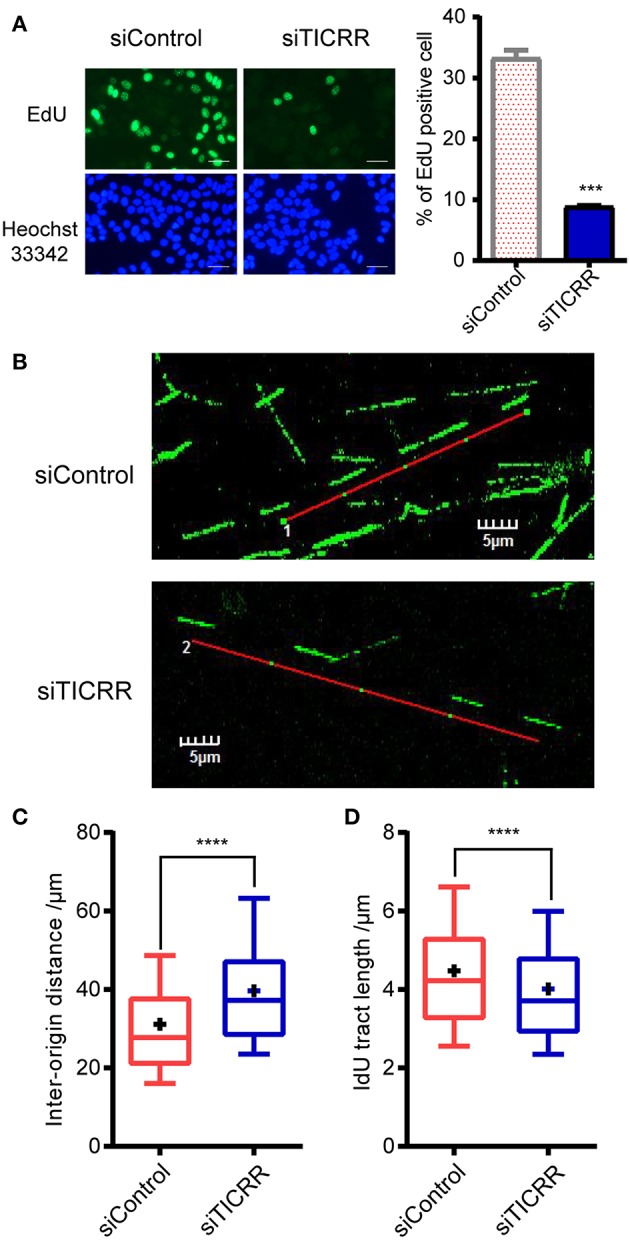
Knockdown of *TICRR* significantly inhibited DNA replication. **(A)** Schematic representation of EdU labeling in MCF7 cells with or without knockdown of *TICRR*. DNA was visualized by Hoechst 33342 staining. Scale bar = 50 μm. The percentage of EdU-positive cells was shown on the right. ***p-value < 1.00 × 10^−3^ two-tailed Student's *t*-test. *N* = 2 biological replicates. **(B–D)** DNA fiber assay in MCF7 cells after transfection of siTICRR and control siRNA. The image of typical DNA fibers was shown in **(B)**. Scale bar = 5 μm. The quantification and the densities of inter-origin distance (IOD) and IdU-labeled tract length were shown in **(C)** and **(D)**. Whiskers indicate 10 and 19th percentiles and center values depict the median and plus depict the mean. ****p < 1.00 × 10^−4^, determined by two-tailed Welch's *t*-test. Experiment number was shown in Table S3.

Given the key role of *TICRR* in DNA replication initiation as well as the observation that alteration of TICRR level changed DNA initiation (12, 13, 21), we proposed that the reduced viability and tumor growth induced by *TICRR* knockdown are attributable to the deficiency of DNA replication. Here we found that *TICRR*-depletion led to significantly reduced percentage of MCF7 cells labeled with EdU (*p* = 2.61 × 10^−4^) (Figure 4A), a molecule combining to DNA during S phase of cell cycle, suggesting DNA synthesis likely to be inhibited. Then, we directly labeled replicating DNA with green fluorescent Iodo-deoxyuridine (IdU) (Figure 4B) and found that the inter-origin distance (IODs) in *TICRR*-depleted cells (mean: 39.63 μm) was significantly lengthened compared with the control (mean: 31.14 μm; *p* = 2.95 × 10^−10^) (Figure 4C; Table S3), indicating that *TICRR* depletion suppressed the initiation of DNA replication origins.

Interestingly, we also observed that the median of IdU tract length in *TICRR* knockdown cells (mean: 4.01 μm) was significantly shortened than that in the control (mean: 4.47 μm; *p* = 1.87 × 10^−11^) (Figure 4D; Table S3), indicating a stalled replication fork progression after *TICRR* depletion. These results collectively suggest that silencing *TICRR* affects not only the initiation of DNA replication but also the progression of replication fork, thus significantly impairing the DNA synthesis.

### *TICRR* Depletion Arrests Cell Cycle at G1 Phase

**Figure 5:**
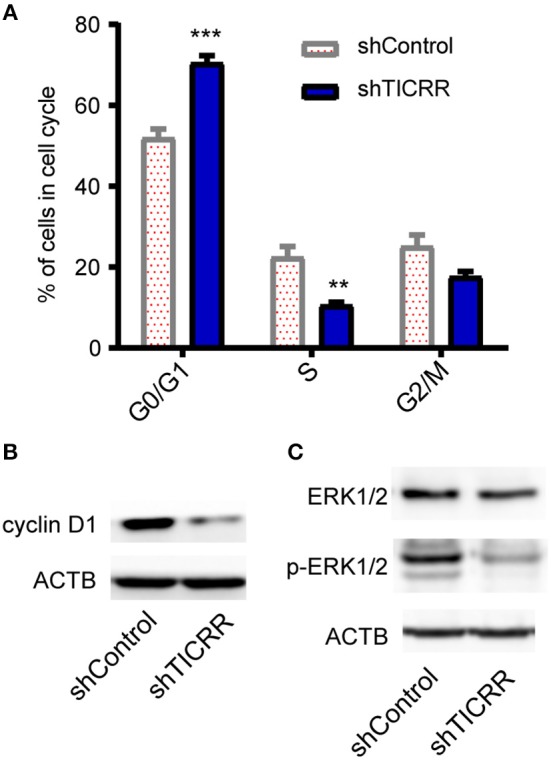
Knockdown of *TICRR* inhibited cell cycle. **(A)** Cell cycle analysis was performed by PI-DNA staining in *TICRR*-knockdown cells compared with control in MCF7 cells. **p-value < 0.01, ***p-value < 1.0 × 10^−3^, two-way ANOVA test. Results were displayed as means ± SEM, *N* = 3 biological replicates. **(B)** cyclin D1 protein level were shown in MCF7 cells. **(C)** ERK1/2 and p-ERK1/2 protein levels were shown in MCF7. *N* = 3 biological replicates.

Since *TICRR* functions to activate the origins from G1/S transition to S-phase (22) and regulate S-phase duration (12), cell cycle analysis was performed in *TICRR* knockdown cells. We found that *TICRR* silencing induced significant arrest of cell cycle at G0/G1-phase in MCF7 cells with increased percentage of G0/G1-phase cells (*p* = 1 × 10^−4^) (Figure 5A; Figure S5A). The results were validated in SKBR3 (*p* = 6.9 × 10^−3^) and MCF10A cells (*p* = 7.8 × 10^−3^) (Figures S5A,B). We also found that *TICRR* depletion decreased the expression of Cyclin D1, which controls cell cycle progression through the G1-S checkpoint, in MCF7 (Figure 5B), SKBR3 cells (Figure S5C), HCC1806 cells and tumors harvested from mice (Figure S5D). Moreover, the level of phosphorylated ERK1/2, a protein involved in the regulation of G1/S transition and DNA replication (23), also decreased in *TICRR* knockdown MCF7 cells (Figure 5C), indicating that cell cycle was interrupted at S phase entry.

### *TICRR* Knockdown Widely Regulates Expression of Cell Cycle Related Genes

**Figure 6:**
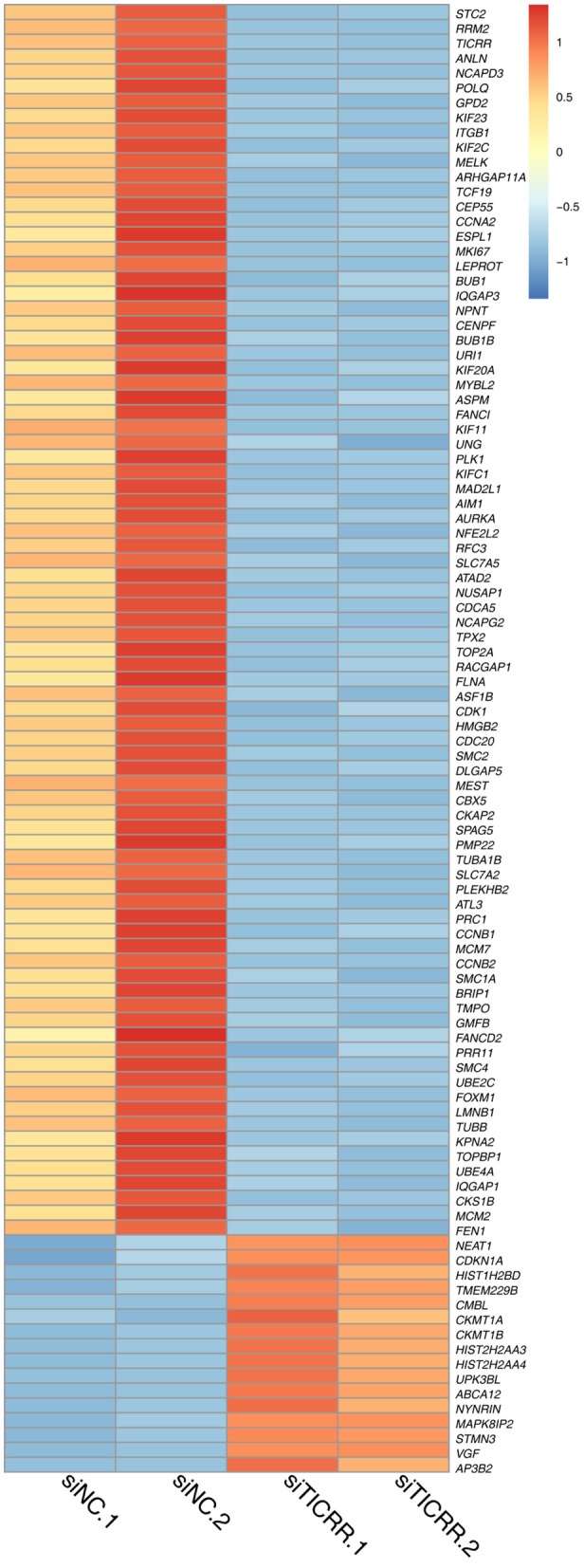
Heatmap of 100 DEGs obtained from two *TICRR*-knockdown samples (siTICRR.1 and siTICRR.2) and two control samples (siNC.1 and siNC.2). Probability of gene expression difference > 0.8 is considered differentially expressed.

**Figure 7:**
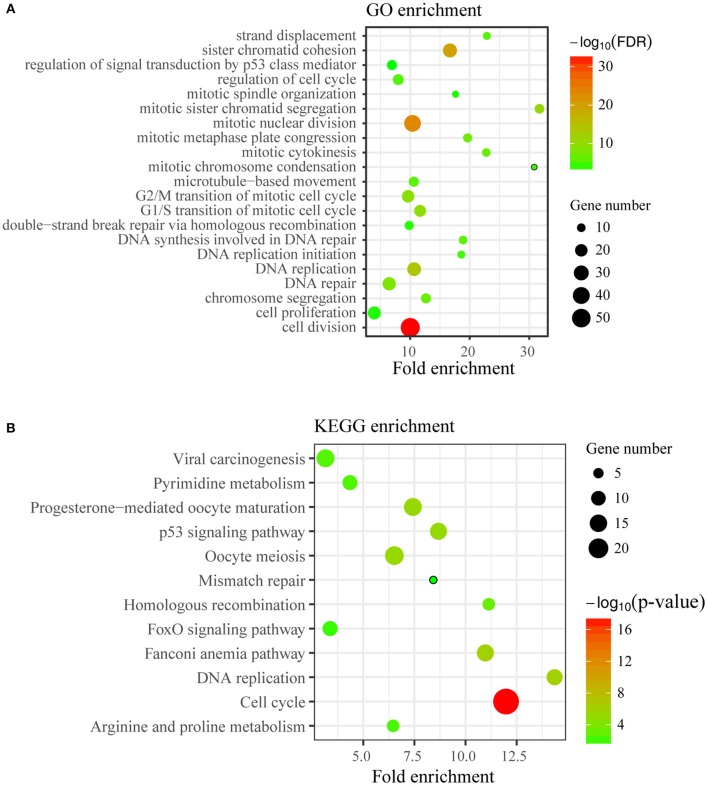
Enrichment analysis of DEGs. **(A)** Top21 of Gene Ontology (GO) enrichment (FDR < 0.05) of differentially expressed genes were displayed. The y-axis represents the Gene Ontology term name and the x-axis represents fold enrichment of matched ontology categories. The circle size represents the gene count of matched term and the circle color showed the FDR. **(B)** KEGG pathway analysis on differentially expressed genes was conducted. The y-axis represents the pathway term name and the x-axis represents fold enrichment. The circle size represents the gene count of matched term and the circle color represents the *p*-value.

To disclose genes and/or pathways involved in the arrestment, we obtained the transcriptomes of MCF7 cells transfected with *TICRR*-specific siRNA and the control after 48 h via RNA-sequencing. For the obtained gene expression data, mRNA levels of several randomly selected genes were estimated and confirmed to be replicable via RT-qPCR (Figure S6A). Our analysis showed that 53 genes were up-regulated and 225 genes down-regulated with fold change >2 (Figure 6; Table S4). As expected, gene ontology (GO) analysis revealed that the DEGs were significantly enriched in cell cycle and its regulation processes, such as cell division, DNA replication, G1/S and G2/M transition of mitotic cell cycle (Figure 7A; Table S5). KEGG pathway analysis also showed cell cycle and DNA replication as the most significant enrichments (Figure 7B; Table S6).

We further analyzed the mRNA levels of genes that directly interact with *TICRR* in DNA replication initiation process and found that *MCM2, MCM5*, and *TOPBP1* expression decreased significantly after *TICRR* silencing (all *P* < 0.05), while *CDC45* and *MCM7* were not changed in MCF7 cells (Figure S6B). However, the genes *CDC45, MCM2, MCM5*, and *MCM7*, were found to be consistently down-regulated in SKBR3 cells and HCC1806 cells (all *P* < 0.05) (Figure S6C). These results altogether showed that the depletion of *TICRR* greatly affected the expression of cell cycle-related genes, especially those involved in DNA replication, and thus led to cell cycle arrest and subsequently inhibition of cell proliferation.

### *TICRR* Knockdown Triggers p53 Signal Pathway

**Figure 8:**
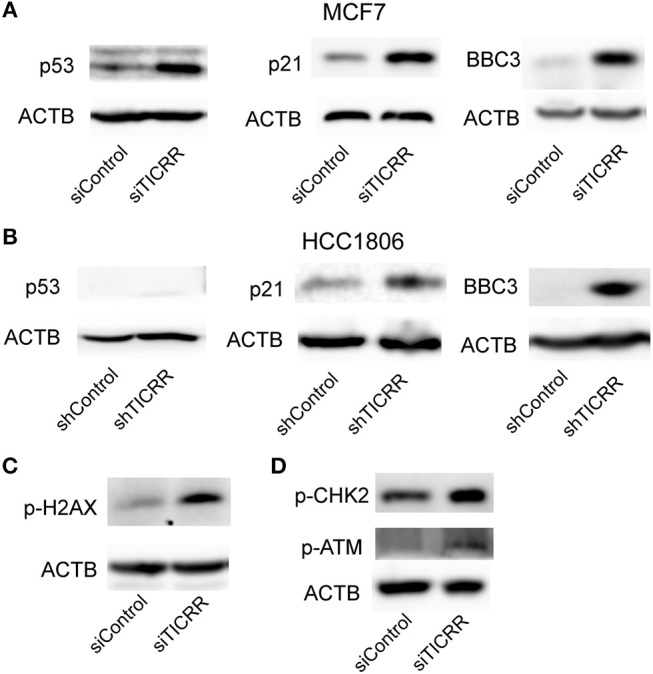
Knockdown of *TICRR* activated ATM/CHK2-dependent p53 signal pathway. **(A)** Protein levels of p53, p21 and BBC3 in control and *TICRR*-knockdown MCF7 cells. *N* = 3 biological replicates. **(B)** Protein levels of p53, p21, and BBC3 in control and *TICRR*-knockdown HCC1806 cells. *N* = 3 biological replicates. **(C)** The p-H2AX protein in MCF7 cancer cells after transfection with siRNA for 8 h. *N* = 3 biological replicates. **(D)** Protein levels of p-CHK2 and p-ATM in control and *TICRR*-knockdown MCF7 cells. *N* = 3 biological replicates.

We also noticed that p53 signal pathway was significantly enriched in KEGG analysis (*p* = 7.25 × 10^−6^) (Figure 7B; Table S6), suggesting the p53 signal pathway likely be activated. The transcriptomic data revealed the downstream genes of p53, such as *CDKN1A, CCNE2, CCNB1/2, CDK1, BID*, and *BBC3*, were altered (Figure S7A), which may contribute to G1/G2 arrest and cell apoptosis (24). We then determined the protein levels of p53 and found that *TICRR* depletion enhanced the expression of p53 in MCF7 cells (Figure 8A). Indeed, at the translation level, CDKN1A/p21, the main regulator of cell cycle from G1 to S transition (25, 26), and the protein regulating cell apoptosis, BBC3 (27), were also up-regulated in *TICRR*-depleted MCF7 cells (Figure 8A). We also found the genes *CCNE2, CCNB1/2, CDK1*, and *BID* to be down-regulated and p21 and BBC3 to be up-regulated in HCC1806 which was reported as a p53-null cell line (Figure S7B and Figure 8B). In SKBR3, a p53 mutant cell line, we also found that knockdown of *TICRR* can increased p21 and BBC3 (Figure S7C), suggesting alternative way may function, such as p73 that can replace p53 to regulate cell proliferation and apoptosis in p53-null cells (28), which have yet to be determined.

### *TICRR* Knockdown Activates DNA Damage Response via ATM/CHK2

As the impaired fork progression of DNA replication can lead to DNA damage (2, 29, 30), the known activator of p53 pathway (25), it is therefore possible that the activation of the p53 pathway in *TICRR*-depleted MCF7 cells is triggered by DNA damage response (DDR). Indeed, we found that the protein level of phosphorylated histone H2AX, a marker of DNA damage (31), was increased in *TICRR* depletion cells (Figure 8C). Further, one of the key kinases involved in DDR, ataxia telangiectasia mutated (ATM), was examined. Our results showed that the level of phosphorylated ATM was up-regulated in *TICRR*-depleted MCF7 cells (Figure 8D). Accordantly, the level of phosphorylated checkpoint kinases 2 (p-CHK2) was also increased (Figure 8D), indicating that both ATM and CHK2 were activated. The activated ATM-dependent p53 signaling induced by *TICRR* knockdown was validated in MCF10A cells (Figure S8A). We also determined but failed to detect the expression of the p-CHK1, the downstream protein of another DDR kinase ATR, no matter in *TICRR*-depletion or in the control cells (Figure S8B). Even under the treatment of cisplatin, a DNA damage inducer, p-CHK1 protein was observed to be increased in the control cells but not in *TICRR* knockdown cells (Figure S8B), suggesting that it is ATM/CHK2, rather than ATR/CHK1, which was activated in *TICRR*-depleted MCF7 cells.

### *TICRR* Expression Significantly Correlates With Cancer Prognosis

**Figure 9:**
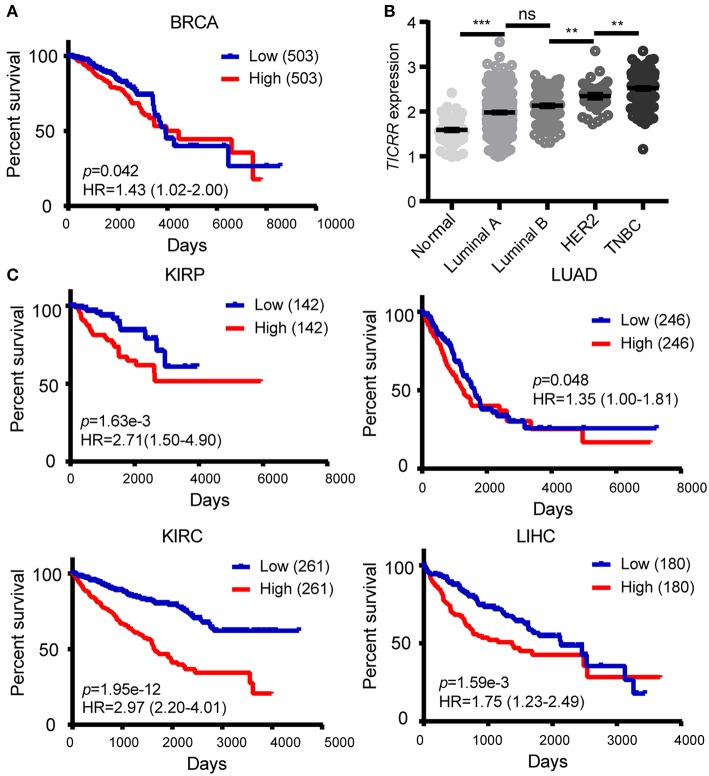
Association of *TICRR* expression with clinical outcomes. **(A)** Kaplan-Meier survival analysis was conducted between *TICRR* expression and overall survival of patients with BRCA using the web-tool OncoLnc. **(B)** Patients with different subtypes of breast cancer showed different expression levels of *TICRR* [log10(1 + FPKM)]. Luminal A, ER/PR-positive and HER2-negative; luminal B, ER/PR-positive and HER2-positve; HER2, ER/PR-negative and HER2-positve; TNBC, ER/PR-negative and HER2-negative. **p < 0.01, ***p < 1.00 × 10^−3^, one-way ANOVA test. Results were displayed as means ± SEM. **(C)** Kaplan-Meier survival analysis was performed between *TICRR* expression and overall survival of patients with KIRC, KIRP, LIHC, and LUAD. Median of *TICRR* mRNA expression was used to group and *p*-value was calculated using log-rank tests. HR, Hazard Ratio.

**Figure 10:**
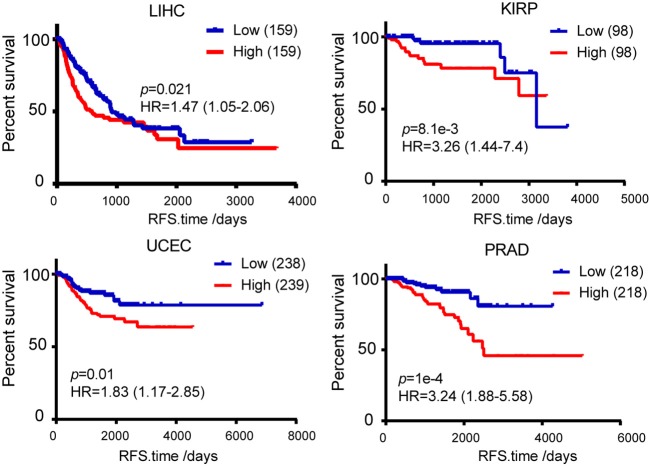
Kaplan-Meier survival analysis was used to analyze the association between *TICRR* expression and patient disease free survival in LIHC, KIRP, UCEC, and PRAD. Median of *TICRR* mRNA expression was used to group and *p*-value was calculated using log-rank tests. HR, Hazard Ratio.

The obtained evidence collectively lends support to a critical role of *TICRR* in tumorigenesis through regulating DNA replication in cancer cells, it is then possible that this gene would have great potential in clinical prognosis. We examined the association between *TICRR* expression and survival of cancer patients using the TCGA clinical data. The result revealed that the patients with higher *TICRR* expression (more than the median value) showing significantly poorer overall survival (OS) than those with lower expression in breast cancer (*p* = 0.042) (Figure 9A). We further determined the association between *TICRR* expression and distinct subtypes of breast cancer, classified by estrogen receptor (ER), progesterone receptor (PR), and human epidermal growth factor receptor 2 (HER2) expression patterns (32, 33). The results showed *TICRR* displayed the highest expression in triple-negative breast cancer (TNBC, ER/PR-negative, and HER2-negative), followed by HER2-positive breast cancer (HER2, ER/PR-negative, and HER2-positive) (Figure 9B), which are both associated with shorter survival time (32). Similar correlations were observed in the other cancer types, including KIRC, KIRP, LIHC, and LUAD (all *P* < 0.05) (Figure 9C). Besides, we also found that higher *TICRR* expression was associated with lower disease free survival (DFS) in LIHC, KIRP, UCEC, and PRAD (all *P* < 0.05) (Figure 10).

## Discussion

Cancer is essentially a disease with uncontrolled cell proliferation. Understanding the mechanisms underlying the capability of cancer cell to sustain unlimited proliferation is the key to find the avenue for therapy of the disease. So far, it is known that such kind of capability could be achieved through several ways, such as increased growth signals, reduced or inhibited growth suppressors, and reprogramming metabolism (34, 35), which facilitates rapid cell growth and division via providing plenty of energy and/or bio-mass (36). Since each cancer cell contains its own genome, it therefore requires a high rate of DNA replication to satisfy their genome propagations and resultant proliferation. Indeed, elements involved in DNA replication, for example cellular dNTP level and the replication machinery, have been suggested to be associated with cancer cell proliferation (37, 38) and as important targets for developing anti-cancer drugs (39, 40). Therefore, discovering the key regulator of DNA replication in cancer cells would help to gain more insight into the mechanisms of tumorigenesis and, most importantly, develop a new avenue for anti-tumor therapy.

In this study, we report for the first time that *TICRR*, previously known as a hub gene in the assembly and activation of DNA helicase, was up-regulated universally in 15 solid tumor types, suggesting it to likely function in tumorigenesis. Indeed, we reveal that *TICRR* depletion strikingly inhibited tumor cell proliferation, migration *in vitro* and tumor growth *in vivo*. Further evidence shows that *TICRR* can significantly affect the initiation of DNA replication, with reduced density of active origins in *TICRR*-depletion cells. Similar results were obtained in *TICRR* over-expressed and knockdown U2OS cells (13, 21). Thus, the high initiation of cancer cell resulted from high expression level of *TICRR* may account for the observations that cancer cells have higher origin activity (41, 42). Different from other reports that no role of *TICRR* in elongation of DNA replication (43, 44), we found that the fork progression was stalled by *TICRR* knockdown. The result is different from other studies reporting that over-expression of TICRR mutant or reduced level of TICRR had no effects on fork rate (12, 21), and was in contrast to a recent study showing that TICRR-knockdown accelerated the fork extension (13). In fact, they also concluded that p53-p21 axis acted as a negative regulator of fork speed. Thus, one reason for the stalled fork progression is up-regulated level of p53 and p21 after *TICRR*-knockdown in MCF7 cells. In addition, knocking *TICRR* down resulted in the down-regulation of transcripts for DNA replication elongation factors, including *POLA2, PRIM1, RFC4, RFC3, DNA2, FEN1, EXO1* (45) (Table S4). Another may be the reduced expression of *RRM1* and *RRM2* along with depletion of *TICRR*, two main subunits of ribonucleotide reductases (RNRs) required for synthesis of dNTP (37, 46). Mild perturbation of RNRs results in redox imbalance which leads to slowdown fork speed and further inhibition of RNRs, resulting in deficient dNTP pool, fork stalling and DNA breaks (47, 48).

TICRR was also identified to interact with TOPBP1 in both S/M and G2/M checkpoints and activate ATR-mediated CHK1 phosphorylation (49, 50). This well explains why CHK1 could not be activated by genetic toxic agents in *TICRR*-depletion cells (Figure S7). Dysregulated DNA replication and abrogation of checkpoint response force cells to enter mitosis with incompletely replicated DNA, thus leading to DNA damage. This activates the ATM/CHK2 DNA damage response, as evidenced by the increased activity of CHK2. Furthermore, the downstream of this response, p53-dependent cell-cycle arrest and apoptotic enhancement to DNA damage (51), are also observed in the *TICRR*-depletion cells. Overall, our results demonstrate that a high level of *TICRR* promotes proliferation of cancer cell through firing more replication origins, while reducing *TICRR* expression triggers the DNA damage-p53 pathway, leading to inhibition of proliferation and enhancement of apoptosis, at least in p53-wild cancer cells (Figure S9).

However, we acknowledge that there are still some limitations in this study. First, p53 is mutated or deleted most frequently in human tumors (52). We found *TICRR* knockdown inhibited cell proliferation and induced cell cycle arrest in HCC1806 (p53-null) as well as SKBR3 (p53 mutant). We also found the same alteration of p53 target genes regardless of p53 status, such as up-regulated p21 and BBC3 in MCF7, HCC1806 and SKBR3 cells (Figures 8A,B and Figure S7C). It has been reported that p73 can replace p53 to mediate cell cycle and apoptosis in p53-null cells (28), so further experiments are needed to verify the hypothesis. In addition, *TICRR* overexpression may further confirm the role of *TICRR* in tumorigenesis.

The critical role of *TICRR* in regulating DNA replication in cancers suggests that this gene has high potential for clinical applications. The depletion of *TICRR* can significantly block breast cancer cell proliferation, migration and tumor growth *in vitro* or *in vivo*, suggesting it to be a promising therapeutic target for breast cancer treatment. Meanwhile, its universal up-regulation in most major solid tumors indicates that this gene could be a common prognostic biomarker. For example, *TICRR* was highly associated with both overall survival and disease free survival of patients with KIRP (*p* = 1.63 × 10^−3^ and 8.1 × 10^−3^, respectively). Importantly, *TICRR* displays the highest expression level in TNBC, with the worst prognosis largely due to the lack of specific prognostic biomarker and effective therapeutic target (53), suggesting it as a good prognostic gene marker in this malignant tumor.

In conclusion, we reveal that *TICRR* is an important oncogenic factor that promotes proliferation of cancer cells by enhancing both initiation and progression of DNA replication. Such a role, together with further evidence from *in vivo/vitro* experiments and clinical analysis, suggesting *TICRR* not only to be a good prognostic biomarker for poor clinical outcome, but also have high potential to be a promising therapeutic target in cancers, especially in kidney and TNBC.

## Author Contributions

QY contributed to designing and conducting the experiment, the data analysis and writing the manuscript. S-YP contributed to analyzing RNA-seq data. HW, X-QC, J-JJ, and K-SG performed the experiment. Y-HH contributed to designing the experiment and writing the manuscript. Q-PK designed the research studies and wrote the paper. All authors reviewed the manuscript.

### Conflict of Interest Statement

The authors declare that the research was conducted in the absence of any commercial or financial relationships that could be construed as a potential conflict of interest.

## Abbreviations

BRCA: breast invasive carcinoma
KIRC: kidney renal clear cell carcinoma
KIRP: kidney renal papillary cell carcinoma
KICH: kidney chromophobe
BLCA: bladder urothelial carcinoma
LUAD: lung adenocarcinoma
LUSC: lung squamous cell carcinoma
COAD: colon adenocarcinoma
HNSC: head and neck squamous cell carcinoma
LIHC: liver hepatocellular carcinoma
UCEC: uterine corpus endometrial carcinoma
PRAD: prostate adenocarcinoma
READ: Rectum adenocarcinoma
STAD: Stomach adenocarcinoma
ESCA: Esophageal carcinoma.
