# Supplementary material for: TICRR Contributes to Tumorigenesis Through Accelerating DNA Replication in Cancers
Source: Front Oncol. 2019 Jun 18;9:516. doi: 10.3389/fonc.2019.00516 (PMC6591320; doi:10.3389/fonc.2019.00516)
Supplement: Supplementary file 1 [file Data_Sheet_1.pdf]

Supplementary materials

TICRR contributes to tumorigenesis through accelerating DNA replication in cancers

Qin Yu, Shao-Yan Pu, Huan Wu, Xiao-Qiong Chen, Jian-Jun Jiang, Kang-Shuyun Gu, Yong-Han He, Qing-Peng Kong

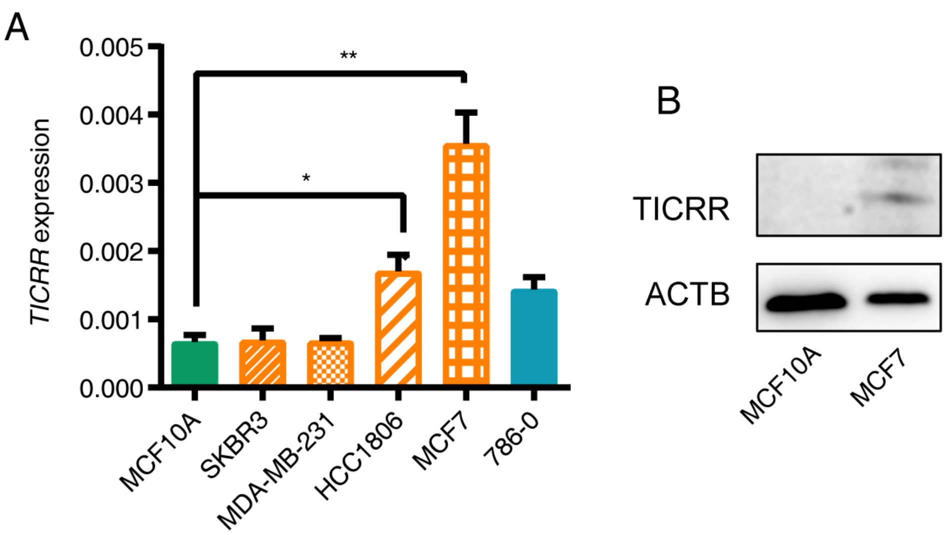

Figure S1. The expression of *TICRR* in cell lines. **(A)** RT-qPCR analysis on mRNA expression of *TICRR* in the immortalized breast epithelial cell lines (MCF10A) and four breast cancer cell (MCF7, SKBR3, HCC1806 and MDA-MB-231), as well as one kidney cancer cell lines (786-0). The level of *TICRR* was normalized to *ACTB*. Data were calculated with $2^{-\Delta CT}$ and were shown as mean ± SEM. * $p < 0.05$, ** $p < 0.01$, two-tailed Student's t test. N = 3. **(B)** The protein level of TICRR in MCF10A and MCF7 cells. N = 3.

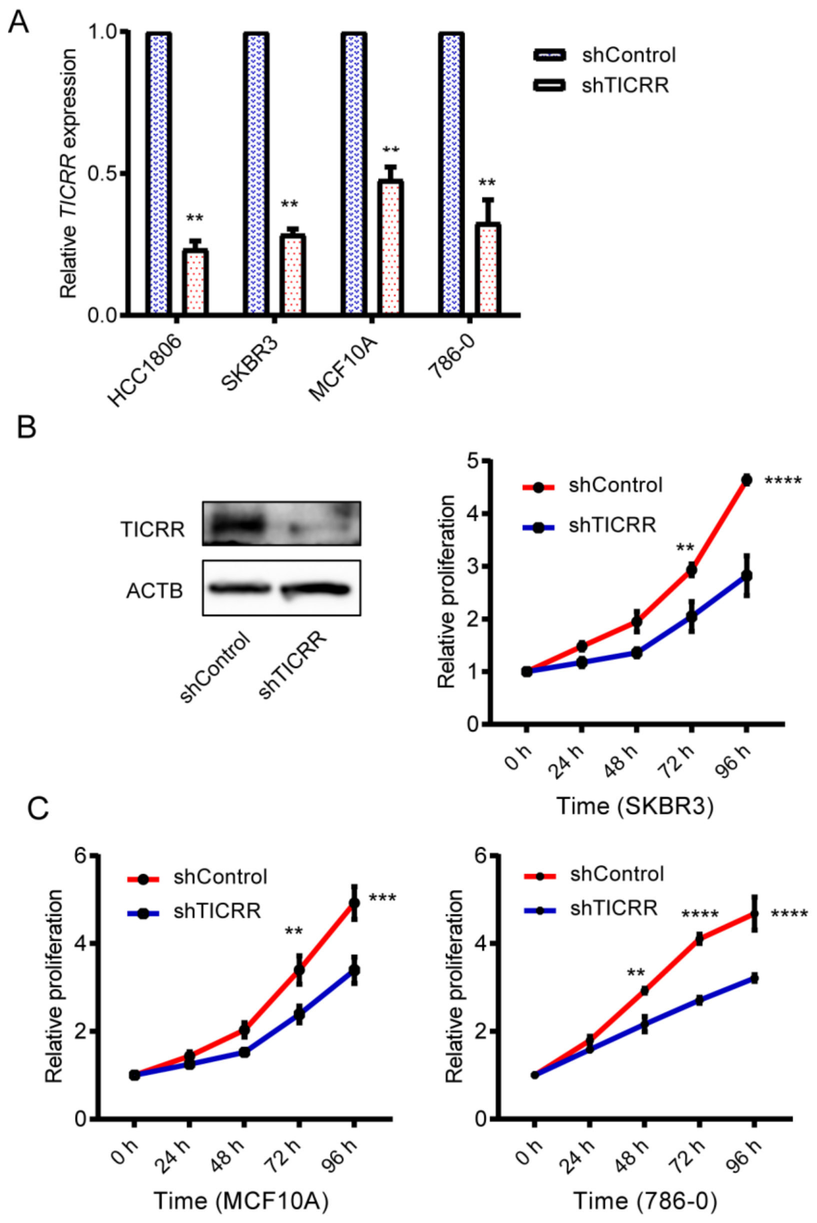

Figure S2. Knockdown of *TICRR* significantly inhibited cell proliferation. **(A)** *TICRR* expression was inhibited in 4 cell lines using shRNA. Efficient knockdown of *TICRR* was revealed by RT-qPCR, and *ACTB* was used as a loading control. ** $p < 0.01$, two-tailed Student's t test. Results were displayed as means ± SEM. N = 3. **(B)** Growth curves in SKBR3. Western blot (left) was performed. Cell viability in 0 H was set at 1. **(C)** Growth curves in MCF10A and 786-0 cell lines. Cell viability in 0 H was set at 1. ** $p < 0.01$, *** $p < 1.0 \times 10^{-3}$, **** $p < 1.0 \times 10^{-4}$, two-way ANOVA test. Results were displayed as means ± SD. N = 3.

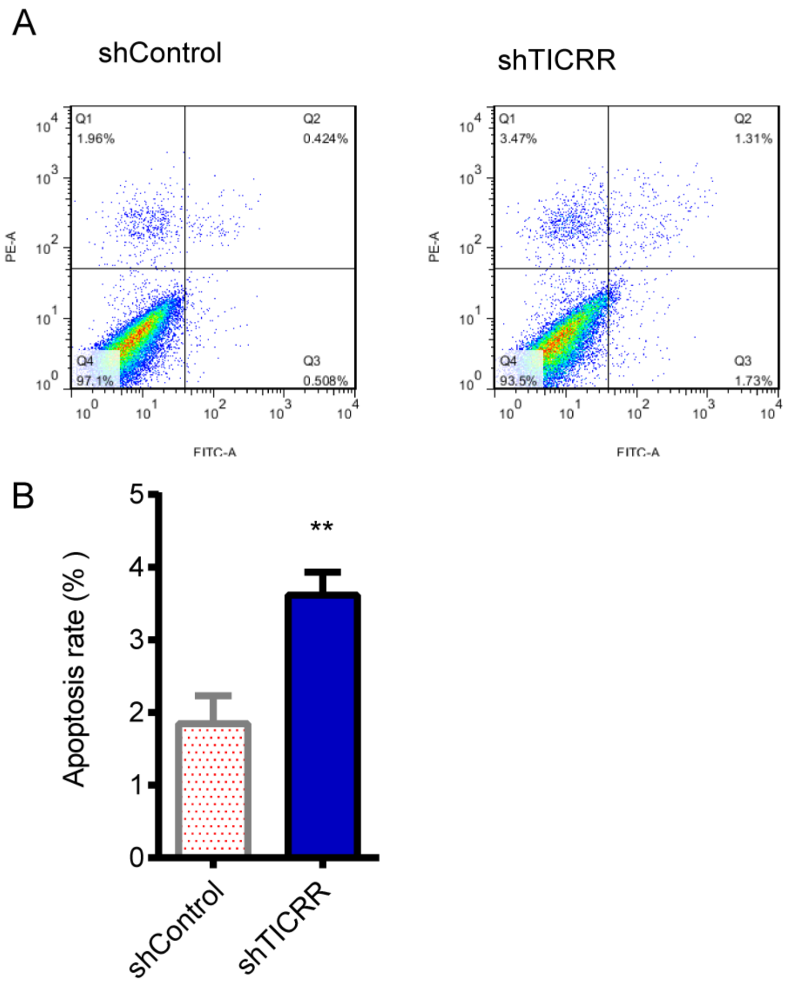

Figure S3. Cell apoptosis was analyzed after *TICRR*-depletion in MCF7 cells. Apoptosis was determined by annexin V-FITC/PI assay and analyzed by flow cytometry. **(A)** Schematic representation. **(B)** Percentage of apoptosis from 4 independent experiments. ** $p < 0.01$, two-tailed Student's t test. Results were displayed as means ± SEM.

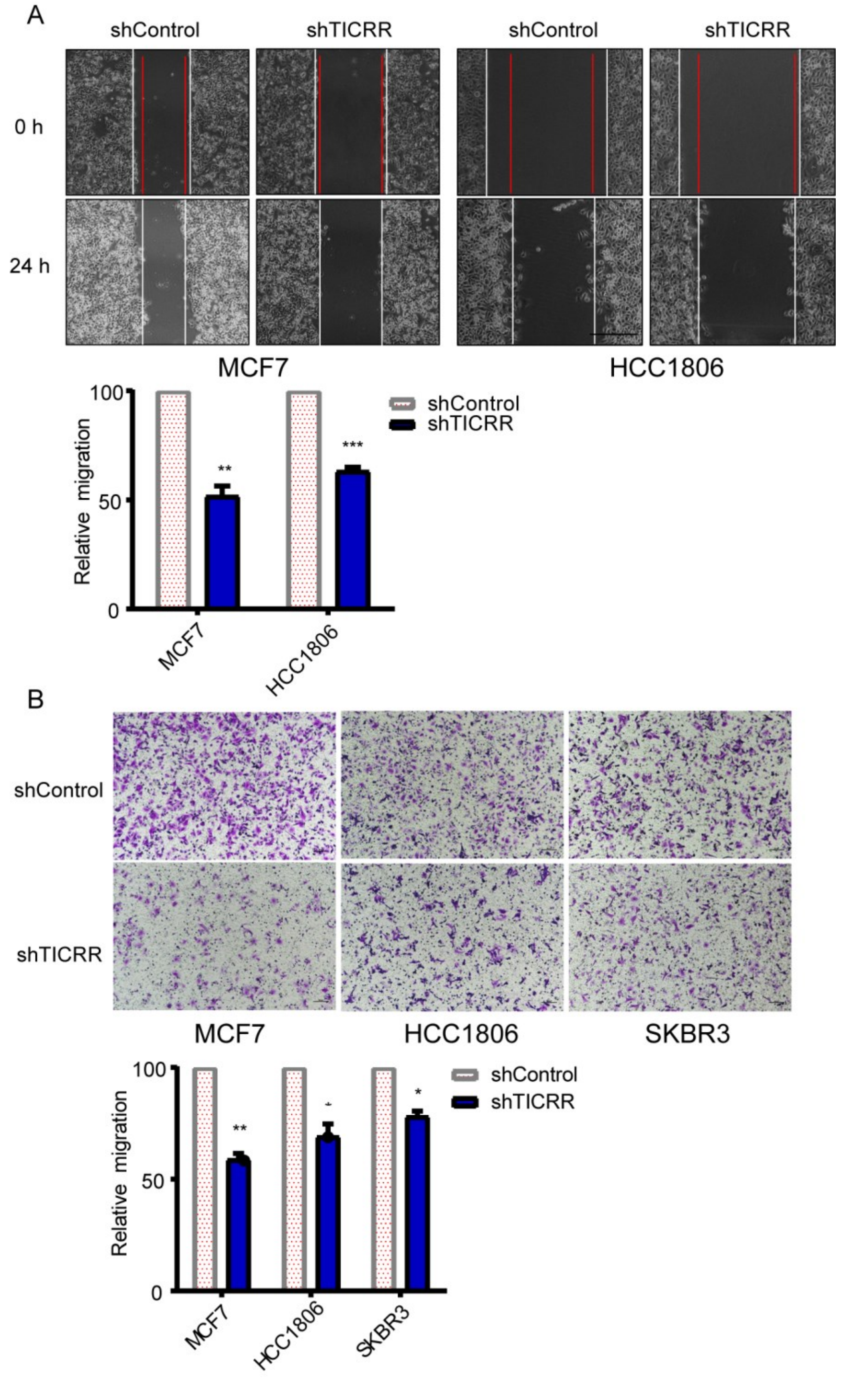

Figure S4. Knockdown of *TICRR* significantly inhibited cell migration *in vitro.* **(A)** *TICRR*-depletion inhibited cell migration in MCF7 and HCC1806 cells examined by wound healing assay. Representative images (up) and relative migration rate (down) were shown. Scale bar = 500 μm. Wound width in control was set at 100. ** $p < 0.01$, *** $p < 1.00 \times 10^{-3}$, two-tailed Student's t test. Results were displayed as means ± SEM, N = 4. **(B)** Knockdown of *TICRR* inhibited the cell migration in MCF7,

HCC1806 and SKBR3 cells examined by transwell migration assay. Quantification of relative migration rate (up) and representative images (down) were displayed. Scale bar = 100 μm. The number of migrated cell in control was set at 100. * $p < 0.05$, ** $p < 0.01$, two-tailed Student's t test. Results were displayed as means ± SEM, N = 3.

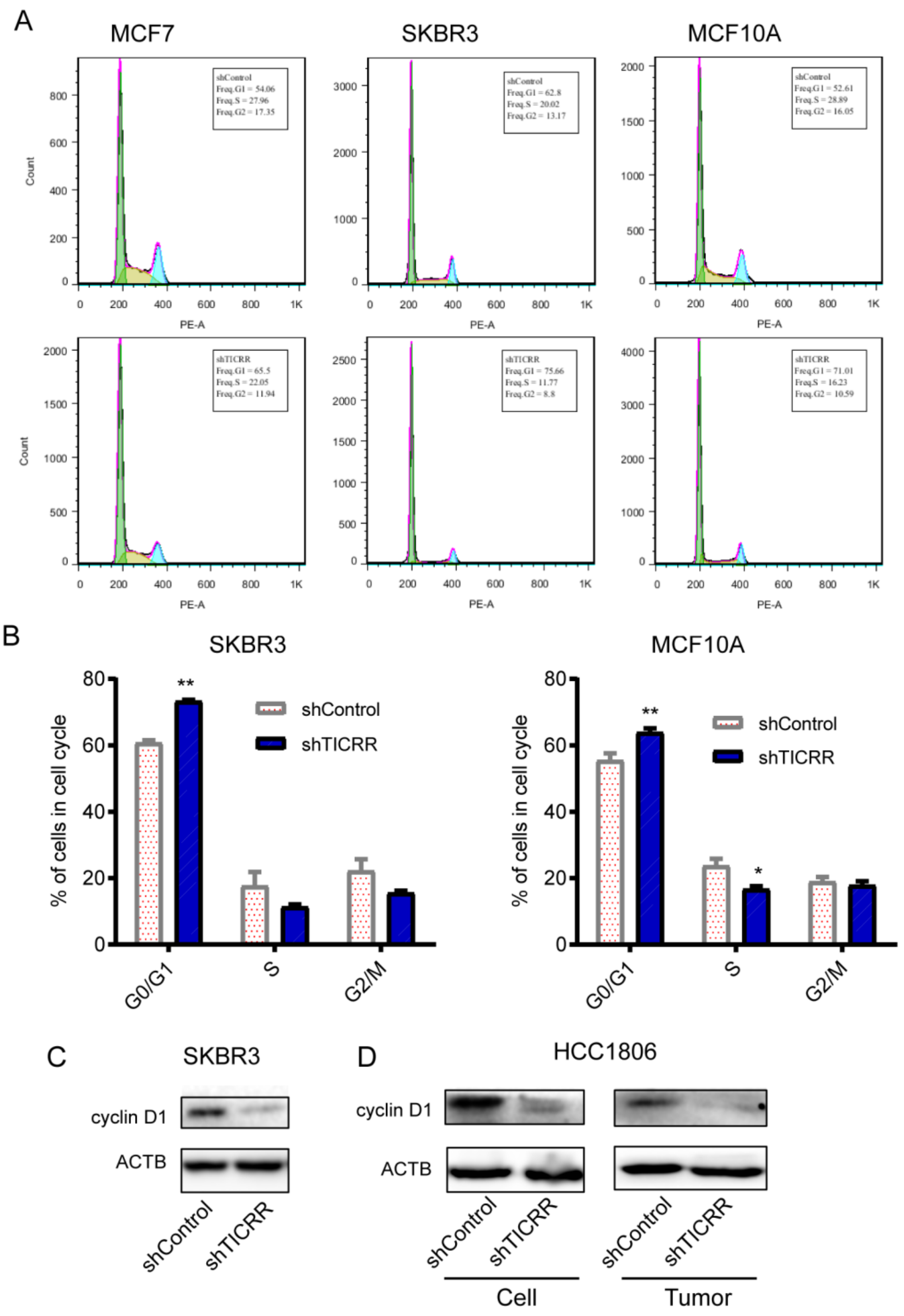

Figure S5. Knockdown of *TICRR* led to cell cycle arrest. **(A)** The representative photos of cell cycle were shown. Cell cycle was analyzed by flow cytometer in MCF7, SKBR3 and MCF10A cells with *TICRR* knockdown. **(B)** Percentages of cells in different phases of the cell cycle were indicated. * $p < 0.05$, ** $p < 0.01$, two-way ANOVA test. Results were displayed as means ± SEM, N = 3. **(C)** cyclin D1 protein level were shown in SKBR3 cells. N = 3 **(D)** cyclin D1 protein level were shown in HCC1806 cells and tumors harvested from mice. N = 2.

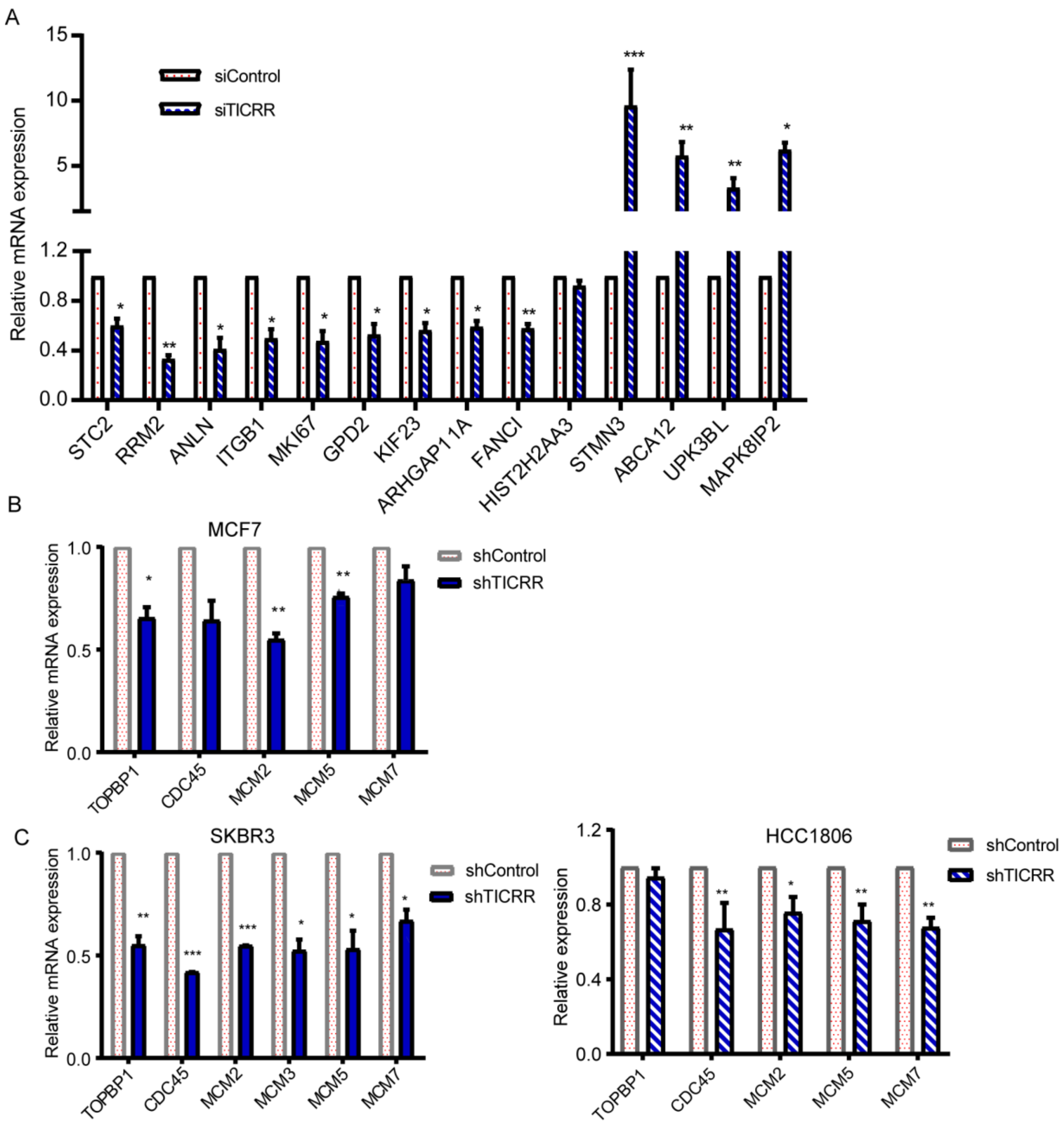

Figure S6. The mRNA expression of several differentially expressed genes and replication initiation gene. **(A)** Validation of several differentially expressed genes using RT-qPCR in MCF7 cells. * $p < 0.05$, ** $p < 0.01$, *** $p < 1.00 \times 10^{-3}$, two-tailed Student's *t* test. Results were displayed as means ± SEM. N = 3. **(B-C)** Expressions of replication initiation genes after *TICRR* knockdown in MCF7 cells **(B)**, SKBR3 and HCC1806 cells **(C)**. * $p < 0.05$, ** $p < 0.01$, *** $p < 1.00 \times 10^{-3}$, two-tailed Student's *t* test. Results were displayed as means ± SEM. N = 3.

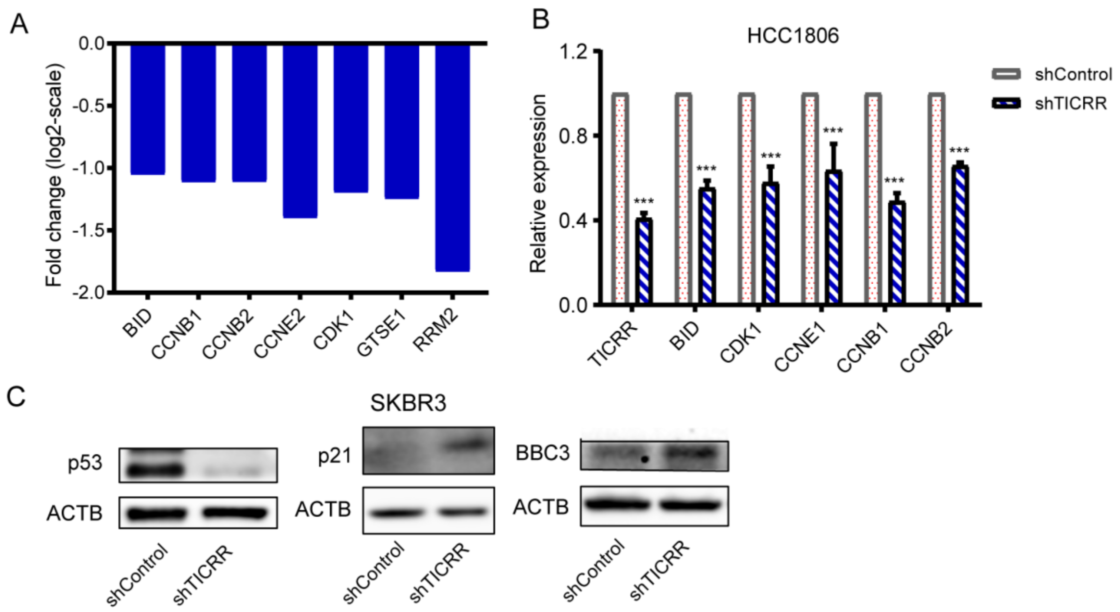

Figure S7. *TICRR*-depletion activated p53 pathway. **(A)** Fold change of significantly p53 target genes in *TICRR*-knockdown MCF7 cells compared to control in RNA-seq data. **(B)** gene verified with RT-qPCR in HCC1806 cells. *** $p < 1.00 \times 10^{-3}$, two-tailed Student's t test. Results were displayed as means ± SEM. N = 3. **(C)** The level of p53, p21 and BBC3 in SKBR3 cells. N = 3.

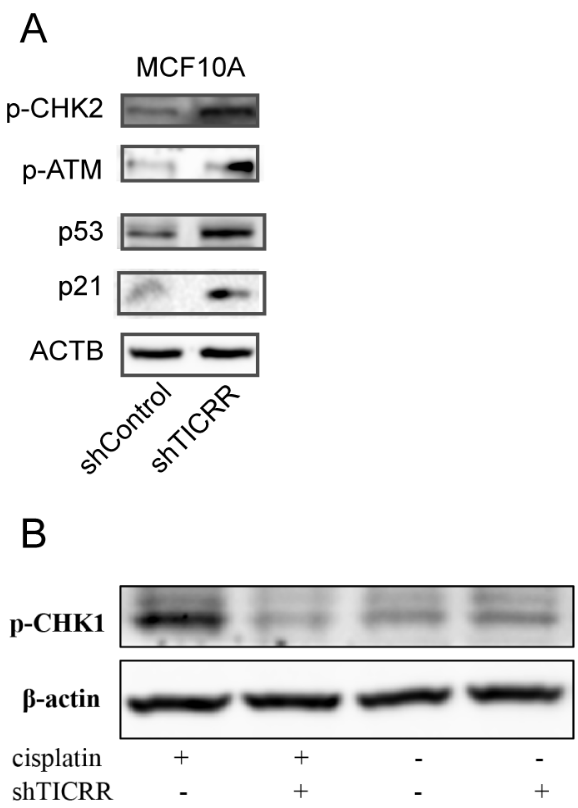

Figure S8. *TICRR*-depletion activated ATM-dependent p53 pathway in MCF10A cells. **(A)** The protein level of phosphorylated CHK2 and ATM, p53 and p21 in *TICRR*-knockdown MCF10A stable cell line. ACTB is loading control, N = 3. **(B)** The level of phosphorylated CHK1 in *TICRR*-knockdown MCF7 cells with or without cisplatin treatment.

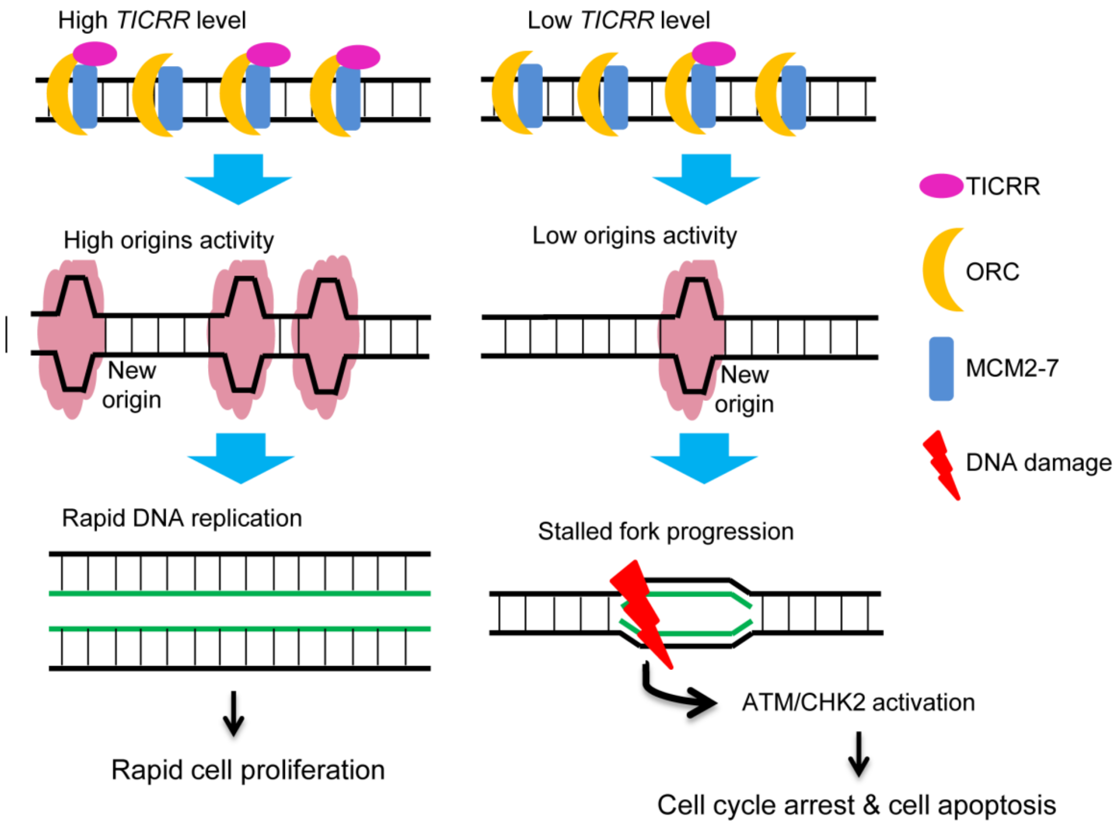

Figure S9. Schematic diagram of our proposed model. High level of *TICRR* in cancer cell promotes rapid DNA replication through activating more origins. Depletion of *TICRR* results in reduced number of activated origin and stalled fork progression, which accumulate DNA damage and activate ATM/CHK2 checkpoint response, leading to cell cycle arrest and cell apoptosis, at least in p53-wild cancer cells.

Table S1. Sequences of siRNA and shRNA of *TICRR*.

**Names**	**Target sequence**
siTICRR#1	CCAACTGATGCCACTTTAA
siTICRR#2	CCGAGACTCCAGTGCATAA
shTICRR	CCAACTGATGCCACTTTAA

Table S2. Primers used in this study.

Primer	Sequence	
	Forward	Reverse
STMN3	CCACCCAAGAAGAAGGACAC	CCTGGCGGCTGAAGTTGT
STC2	GCTCGGCTTCCTCCTGTA	AGGGATGGTGCCTCAAAT
RRM2	CTGGCTGCTCGCTCTGCTT	CGGCGTGTTCTCCTTGTCG
ANLN	TGATGATGCGTCTTTGGT	CTCCCTTGGATGGAACTG
ABCA12	GGAAAACTTTGAAGATGC	TAAATCGTATTGTGGACTGC
UPK3BL	TCACCCTGTCCACCTTCA	CTGGCCTCTGGTCTGGTAC
ITGB1	AATGTAACCAACCGTAGC	GGTCAATGGGATAGTCTTC
MKI67	TTGGTGGGCACCTAAGAC	TGATGGTTGAGGCTGTTC
GPD2	GAACCTTGCCATTGCTCT	CTGCGTCTTTATCATCCATT
IST2H2AA3	GTGGCAAGCAAGGAGGCA	CTGGAGGTGACGAGGGATG
APK8IP2	ACGATGAGGACGAGGAAGAG	GACGCACCAGGTCAAAGC
KIF23	GCTGCTTCCTCGTTGTTT	CTTTGGATTGGGCATAGC
RHGAP11A	CCTCCTTGTGATATTGCG	AAGATTGCTGCTGTCCAT
CCNE1	ACTCAACGTGCAAGCCTCG	GCTCAAGAAAGTGCTGATCCC
FANCI	CAGGTCCTCAACAGGGTTGT	CAGTCTGAAGGGGCAGAAAG
CDC45	CTTGAAGTTCCCGCCTATGAAG	GCATGGTTTGCTCCACTATCTC
TOPBP1	TTCAGCAACTCACAGTTAAGCA	GGCACACTCATACTTCTGACC
MCM2	ATGATCGAGAGCATCGAGAACC	GCCAAGTCCTCATAGTTCACCA
MCM3	GGCCTCCATTGATGCTACCTA	ACTTTGGGACGAACTAGAGAACA
MCM5	AGCATTCGTAGCCTGAAGTCG	CGGCACTGGATAGAGATGCG
MCM7	CCTACCAGCCGATCCAGTCT	CCTCCTGAGCGGTTGGTTT
TICRR	CACGGGAGACGAAGAGGT	CTGGAACAGCAGCGGAGA
ACTB	AGAGCTACGAGCTGCCTGAC	AGCACTGTGTTGGCGTACAG
CCNB1	AACTTTCGCCTGAGCCTATTTT	TTGGTCTGACTGCTTGCTCTT
CCNB2	TTGGCTGGTACAAGTCCACTC	TGGGAACTGGTATAAGCATTGTC
CDK1	AAACTACAGGTCAAGTGGTAGCC	TCCTGCATAAGCACATCCTGA
BID	ATGGACCGTAGCATCCCTCC	GTAGGTGCGTAGGTTCTGGT
BBC3	GACCTCAACGCACAGTACGAG	AGGAGTCCCATGATGAGATTGT

Table S3. DNA fiber spread analysis.

MCF7 cells	Total fibers number	Fiber analyzed	Experiments	Median µm	Mean µm	Std. Error	*p*-value
siControl	1065	IdU	4	4.22	4.47	0.05	1.87E-11
siTICRR	1330	IdU	4	3.71	4.01	0.04	
siControl	261	IODs	3	27.77	31.14	0.92	2.95E-10
siTICRR	263	IODs	3	37.20	39.63	0.95	

P-values were calculated using two-tailed Welch's *t* test using R software.

Table S4. The gene list with significant difference.

Symbol	EntrezID	log2(E_{TICRR}/E_{NC})	Probability
VGF	7425	3.027198739	0.9896134
STMN3	50861	2.444297031	0.9792804
STC2	8614	-1.851578944	0.9572492
RRM2	6241	-1.829906641	0.9552415
ANLN	54443	-1.783202225	0.9525645
ABCA12	26154	1.820268368	0.9471839
UPK3BL	100134938	1.720531062	0.9431149
ITGB1	3688	-1.526205425	0.941589
MKI67	4288	-1.450804227	0.9406789
GPD2	2820	-1.57436361	0.9389924
HIST2H2AA3	8337	1.545128382	0.9381358
HIST2H2AA4	723790	1.545128382	0.9381358
MAPK8IP2	23542	2.293622726	0.937413
KIF23	9493	-1.561551996	0.9365564
ARHGAP11A	9824	-1.47553044	0.9330496
AP3B2	8120	3.192457215	0.9324874
MYBL2	4605	-1.287159688	0.9316843
CENPF	1063	-1.347119005	0.931604
FANCI	55215	-1.278564176	0.9310954
ATAD2	29028	-1.225129117	0.9310151
FLNA	2316	-1.199202755	0.9307206
SLC7A5	8140	-1.227219228	0.9305868
CCNA2	890	-1.456106516	0.929864
NPNT	255743	-1.386916541	0.9296231
TOP2A	7153	-1.204810995	0.9293019
TUBA1B	10376	-1.155825089	0.9289003
TPX2	22974	-1.206111081	0.92882
HMGB2	3148	-1.19433798	0.9286862
AURKA	6790	-1.249086948	0.9286862
CBX5	23468	-1.171435655	0.9285523
NYNRIN	57523	1.899461401	0.9279901
SLC7A2	6542	-1.153023202	0.927428
NCAPG2	54892	-1.206830949	0.9273477
KIF20A	10112	-1.292537236	0.9270264
NCAPD3	23310	-1.648263657	0.9259021
CEP55	55165	-1.460358265	0.9255274
RACGAP1	29127	-1.203562972	0.9254738
MELK	9833	-1.478468053	0.9254738
CDKN1A	1026	1.10757336	0.9253935
BUB1	699	-1.403212189	0.9252329
MCM7	4176	-1.111199031	0.9251794
NEAT1	283131	1.074987939	0.9250187

CMBL	134147	1.346573375	0.9247778
TMPO	7112	-1.097493299	0.9244833
TUBB	203068	-1.038317498	0.9242424
BRIP1	83990	-1.107766665	0.9241889
KIF2C	11004	-1.514514684	0.9241086
KPNA2	3838	-1.030846927	0.9235196
PRC1	9055	-1.11170337	0.9235196
ESPL1	9700	-1.453780294	0.9233858
ASPM	259266	-1.279739942	0.9233858
PLK1	5347	-1.254519137	0.9233858
IQGAP3	128239	-1.398409537	0.9233055
SMC1A	8243	-1.108121394	0.9231181
CCNB1	891	-1.111415592	0.9230913
SMC4	10051	-1.060151102	0.9229307
LEPROT	54741	-1.405541827	0.9222079
IQGAP1	8826	-1.009724038	0.9217796
LMNB1	4001	-1.040951041	0.9215387
SMC2	10592	-1.190431319	0.921271
KIF11	3832	-1.268077067	0.9206285
RFC3	5983	-1.228374482	0.9206018
CKMT1B	1159	1.487669996	0.9202002
PRR11	55771	-1.06038079	0.9202002
POLQ	10721	-1.6181815	0.9194507
CKAP2	26586	-1.168524199	0.919183
AIM1	202	-1.252255361	0.9187815
GMFB	2764	-1.080856597	0.9185673
DLGAP5	9787	-1.185822281	0.9184602
UNG	7374	-1.266129079	0.9183264
CDK1	983	-1.194342535	0.9176571
NUSAP1	51203	-1.222937239	0.9173894
PLEKHB2	55041	-1.147561107	0.9171485
MCM2	4171	-1.006980126	0.9168005
NFE2L2	4780	-1.233327973	0.9165061
CDCA5	113130	-1.213338177	0.9163454
HIST1H2BD	3017	1.161030007	0.9163454
TICRR	90381	-1.801559786	0.9162384
URI1	8725	-1.307086411	0.9157833
FOXM1	2305	-1.057378417	0.9155959
CDC20	991	-1.191459674	0.9154353
MAD2L1	4085	-1.252721447	0.9150337
TOPBP1	11073	-1.021411646	0.9141503
CKMT1A	548596	1.473963218	0.9135882
ATL3	25923	-1.128243594	0.9132134
TCF19	6941	-1.463044212	0.9132134

BUB1B	701	-1.307332859	0.9128654
TMEM229B	161145	1.30810194	0.9127316
CKS1B	1163	-1.008970073	0.9119552
PMP22	5376	-1.158615679	0.9119552
FEN1	2237	-1.00677446	0.9116875
SPAG5	10615	-1.167093983	0.9115269
MEST	4232	-1.178212088	0.9106971
FANCD2	2177	-1.064438763	0.9101349
UBE2C	11065	-1.058954601	0.9099475
KIFC1	3833	-1.254244062	0.9097334
UBE4A	9354	-1.020429739	0.9097066
CCNB2	9133	-1.108720378	0.9092248
ASF1B	55723	-1.195637543	0.9091444
ALDH4A1	8659	1.109761081	0.9089838
RRM1	6240	-1.010739131	0.9087697
MUC1	4582	1.070327127	0.9087697
FAM72C	554282	-1.348193986	0.908502
UHRF1	29128	-1.063298976	0.9084484
CIT	11113	-1.320131706	0.9083681
HMMR	3161	-1.118522969	0.9078595
PKMYT1	9088	-1.359807263	0.9076721
UBE2T	29089	-1.411152758	0.9075383
NCAPG	64151	-1.363079844	0.90711
FANCA	2175	-1.276286658	0.9066817
TRIP13	9319	-1.393608338	0.9061195
EPAS1	2034	-1.584835557	0.9058786
KIF4A	24137	-1.153044564	0.9056109
CDCA4	55038	-1.276278604	0.9049149
CASC5	57082	-1.430764167	0.9043259
CENPE	1062	-1.240580101	0.9042992
HJURP	55355	-1.437098313	0.9040583
CBFB	865	-1.283650111	0.9037638
MCM10	55388	-1.361234965	0.9034693
KRT13	3860	-1.895166878	0.9033622
ASCL1	429	2.505603319	0.9030946
BIRC5	332	-1.164814001	0.9027733
CENPU	79682	-1.257139232	0.9026395
NCAPH	23397	-1.31508566	0.9021308
NDC1	55706	-1.209261409	0.9017293
CCNE2	9134	-1.399389263	0.9015955
ZAK	51776	-1.267141026	0.9015152
CAB39L	81617	1.521143956	0.9004711
DEPDC1B	55789	-1.376326196	0.9003641
SYP	6855	3.187059749	0.8998287

CDT1	81620	-1.229724217	0.8996145
SKA3	221150	-1.271970452	0.8988114
KIAA1524	57650	-1.213999909	0.8984634
ZNF367	195828	-1.231938326	0.8983028
CDCA8	55143	-1.13691416	0.8980351
FILIP1L	11259	-1.351637607	0.8973123
NUBP1	4682	-1.426405503	0.8950102
FAM83D	81610	-1.28662109	0.8948228
SGOL1	151648	-1.439684201	0.8944748
KNTC1	9735	-1.057512001	0.894448
KIF20B	9585	-1.107248492	0.8938055
BNIPL	149428	1.946866121	0.8934843
WDHD1	11169	-1.026218383	0.8933237
PBK	55872	-1.248951272	0.8928151
C11orf82	220042	-1.623767413	0.8924403
PLK4	10733	-1.351284946	0.8915837
SLC16A7	9194	-1.03231104	0.8907806
TTK	7272	-1.282424861	0.8907538
POLR3K	51728	-1.108820769	0.8906468
PPM1E	22843	1.004996489	0.8904058
KIF15	56992	-1.476109689	0.8895224
BLM	641	-1.315364157	0.8892815
SLC25A43	203427	-1.192498448	0.8889067
KIF14	9928	-1.143902129	0.8887997
BRCA2	675	-1.191901812	0.8887193
CDKN3	1033	-1.135890615	0.8883446
MASTL	84930	-1.098171577	0.8881304
PTPRN2	5799	3.689035991	0.887836
E2F1	1869	-1.261757244	0.886203
SPC25	57405	-1.299313487	0.885507
E2F7	144455	-1.328811914	0.8848913
BARD1	580	-1.203765584	0.8847039
APPL1	26060	-1.056814689	0.8843024
RECQL	5965	-1.193280541	0.8840882
AURKB	9212	-1.224702811	0.883767
PTTG1	9232	-1.018411458	0.8835796
PSRC1	84722	-1.120265148	0.8831513
POLA2	23649	-1.040705695	0.8830175
HPS3	84343	-1.00272223	0.8797783
PPFIA3	8541	1.194618878	0.8794571
CLDND1	56650	-1.09579632	0.8793768
SPDL1	54908	-1.078539722	0.8793768
UNC13A	23025	5.180565578	0.878895
SGOL2	151246	-1.007793066	0.8784399

LOC100093631	100093631	-1.007245071	0.8784399
CLSPN	63967	-1.253876378	0.8780116
SHCBP1	79801	-1.269612272	0.8776636
HIST1H4H	8365	1.854858099	0.8768605
CENPJ	55835	-1.253312555	0.8757362
TROAP	10024	-1.116452077	0.8756826
BID	637	-1.050513115	0.8743441
BTBD3	22903	-1.121593106	0.8738355
MZT1	440145	-1.231428978	0.873782
GMNN	51053	-1.031805796	0.8733269
CDC25A	993	-1.694419899	0.8728183
MYBL1	4603	-1.065822388	0.8721223
DIAPH3	81624	-1.349058552	0.8718814
NDC80	10403	-1.120015748	0.8716137
RAD54B	25788	-1.042210793	0.8711318
RFC4	5984	-1.003233516	0.8708641
CENPK	64105	-1.375188749	0.8697398
GDAP1	54332	1.118825349	0.8695792
CKAP2L	150468	-1.623539533	0.8694453
MAOB	4129	1.915248234	0.8692312
ARNTL2	56938	-1.846290585	0.8690706
GTSE1	51512	-1.246371711	0.8685352
RPS6KA4	8986	-1.03858187	0.868321
PBXIP1	57326	-1.124000712	0.8661795
RMDN3	55177	-1.100488412	0.8660188
OSTM1	28962	-1.085858568	0.8646536
DCLK1	9201	1.137667662	0.8643324
NUF2	83540	-1.146298774	0.8640111
GSTCD	79807	-1.621211386	0.8636364
TGFBI	7045	-1.558462131	0.8632884
EXO1	9156	-1.495067227	0.863101
FAM111B	374393	-1.216229986	0.8625656
DCK	1633	-1.10931535	0.8624585
PCLO	27445	1.175654362	0.8623514
CENPA	1058	-1.210509453	0.860772
LRP8	7804	-1.002045581	0.8603705
RAD54L	8438	-1.438326136	0.8600225
MAPK8IP1	9479	1.073582631	0.8597013
CDC6	990	-1.064057314	0.8591659
DSCC1	79075	-1.297932086	0.8588982
SLC7A8	23428	1.739937161	0.8588446
PRODH	5625	1.626492074	0.8584163
ERCC6L	54821	-1.305298966	0.8547221
CDCA3	83461	-1.289585455	0.8538655

SGK3	23678	-1.15523001	0.8534104
FIGNL1	63979	-1.062432896	0.8507335
PGM2L1	283209	1.11789154	0.8506799
CDC45	8318	-1.095414413	0.8496627
CCSAP	126731	-1.560945876	0.8495556
WDR62	284403	-1.102116682	0.8490738
DEPTOR	64798	1.262810923	0.8479495
DISP2	85455	2.515335935	0.8458614
CEP78	84131	-1.009956897	0.8454599
CENPQ	55166	-1.440820214	0.8451654
GGH	8836	-1.042157144	0.8451654
INIP	58493	-1.144151303	0.8439073
PRIM1	5557	-1.057377414	0.8436931
FAM64A	54478	-1.337782399	0.842194
ACKR3	57007	-1.173241787	0.842194
CENPH	64946	-1.024607858	0.8418996
RAD51AP1	10635	-1.47175189	0.8417657
DDX12P	440081	-1.494628091	0.8416586
C1orf112	55732	-1.333578455	0.8409894
FDXR	2232	1.044073284	0.8404808
FAM72B	653820	-1.301888211	0.8398651
BRCA1	672	-1.022307662	0.8386872
RAD51	5888	-1.185145996	0.8386069
FBXO27	126433	1.239586133	0.834645
SMIM13	221710	-1.002801971	0.8343773
DNA2	1763	-1.198474806	0.8340026
SPIN4	139886	-1.054268308	0.8337616
LYPD6	130574	-1.168162393	0.8331192
MAPK11	5600	1.672655235	0.8312721
UTP6	55813	-1.077625191	0.8307099
DTL	51514	-1.092887298	0.8306296
IL6ST	3572	-1.218808211	0.8298265
TMEM198	130612	2.19144724	0.8295321
SFXN5	94097	-1.008517905	0.8290502
HIST1H2AE	3012	1.319824807	0.8288896
SPATA18	132671	1.111736551	0.8278724
SPTBN4	57731	3.298791596	0.8261859
GPR137B	7107	-1.032254584	0.8251954
NEK2	4751	-1.127324477	0.8240443
ESCO2	157570	-1.487480062	0.8220902
CENPI	2491	-1.29403685	0.8220634
BSN	8927	2.499758931	0.8209391
RTN2	6253	1.265041534	0.8206446
RAB8B	51762	-1.137218607	0.820136

KIF18A	81930	-1.164875394	0.8200021
ARL13B	200894	-1.020483735	0.8196809
CRAT	1384	2.092757141	0.8196541
KIF18B	146909	-1.453627089	0.8162544
HIST1H2BC	8347	1.64936111	0.814327
RUNDC3A	10900	3.914564523	0.8140058
POLR2J3	548644	1.105621433	0.8138184
MMS22L	253714	-1.286717333	0.8123461
BBC3	27113	1.338789377	0.8117839
ACTA2	59	2.979040381	0.8113021
KATNAL1	84056	-1.450046985	0.8098833
CAPS	828	1.094975591	0.8096691
RTKN2	219790	-1.245624728	0.809107
RBL1	5933	-1.090140992	0.8081433
SLC9A6	10479	-1.155857638	0.8044759
C4orf46	201725	-1.150217603	0.8018524

Table S5. Gene Ontology analysis of differentially expressed genes.

Term	Count	*p*-value	FDR
GO:0051301~cell division	53	8.27E-37	1.34E-33
GO:0007067~mitotic nuclear division	39	1.99E-27	3.22E-24
GO:0007062~sister chromatid cohesion	26	1.63E-23	2.64E-20
GO:0006260~DNA replication	25	9.22E-18	1.49E-14
GO:0000070~mitotic sister chromatid segregation	12	2.70E-14	4.37E-11
GO:0000082~G1/S transition of mitotic cell cycle	18	1.90E-13	3.08E-10
GO:0000086~G2/M transition of mitotic cell cycle	20	2.22E-13	3.59E-10
GO:0006281~DNA repair	23	8.98E-12	1.45E-08
GO:0007080~mitotic metaphase plate congression	11	1.23E-10	1.99E-07
GO:0000281~mitotic cytokinesis	10	2.67E-10	4.33E-07
GO:0007059~chromosome segregation	13	3.64E-10	5.89E-07
GO:0000731~DNA synthesis involved in DNA repair	10	1.74E-09	2.82E-06
GO:0000732~strand displacement	9	2.94E-09	4.76E-06
GO:0007018~microtubule-based movement	13	2.97E-09	4.81E-06
GO:0051726~regulation of cell cycle	15	5.41E-09	8.76E-06
GO:0006270~DNA replication initiation	9	1.83E-08	2.96E-05
GO:0007076~mitotic chromosome condensation	7	4.92E-08	7.98E-05
GO:0000724~double-strand break repair via homologous recombination	11	1.56E-07	2.52E-04
GO:0008283~cell proliferation	22	1.74E-07	2.82E-04
GO:0007052~mitotic spindle organization	8	2.46E-07	3.98E-04
GO:1901796~regulation of signal transduction by p53 class mediator	13	3.77E-07	6.11E-04
GO:0000722~telomere maintenance via recombination	8	3.96E-07	6.41E-04
GO:0051310~metaphase plate congression	6	5.42E-07	8.78E-04
GO:0006974~cellular response to DNA damage stimulus	16	6.22E-07	0.0010
GO:0090307~mitotic spindle assembly	8	9.33E-07	0.0015
GO:0000079~regulation of cyclin-dependent protein serine/threonine kinase activity	8	1.65E-06	0.0027
GO:0007051~spindle organization	6	2.85E-06	0.0046
GO:0031145~anaphase-promoting complex-dependent catabolic process	10	2.85E-06	0.0046
GO:0034080~CENP-A containing nucleosome assembly	8	3.29E-06	0.0053
GO:0006977~DNA damage response, signal transduction by p53 class mediator resulting in cell cycle arrest	9	3.98E-06	0.0064
GO:0007049~cell cycle	15	5.47E-06	0.0089
GO:0000910~cytokinesis	8	7.05E-06	0.0114
GO:0007094~mitotic spindle assembly checkpoint	6	9.62E-06	0.0156
GO:0034501~protein localization to kinetochore	5	9.84E-06	0.0159
GO:0007019~microtubule depolymerization	5	9.84E-06	0.0159
GO:0032467~positive regulation of cytokinesis	7	1.73E-05	0.0281
GO:0042787~protein ubiquitination involved in ubiquitin-dependent protein catabolic process	12	2.07E-05	0.0336

Table S6. Kyoto encyclopedia of genes and genomes (KEGG) pathway analysis of differentially expressed genes.

**Term**	**Count**	***p*-value**
hsa04110:Cell cycle	23	4.80E-18
hsa03030:DNA replication	8	9.48E-07
hsa03460:Fanconi anemia pathway	9	1.18E-06
hsa04114:Oocyte meiosis	11	5.66E-06
hsa04914:Progesterone-mediated oocyte maturation	10	6.34E-06
hsa04115:p53 signaling pathway	9	7.25E-06
hsa03440:Homologous recombination	5	9.25E-04
hsa05203:Viral carcinogenesis	10	0.0041
hsa00240:Pyrimidine metabolism	7	0.0051
hsa00330:Arginine and proline metabolism	5	0.0070
hsa04068:FoxO signaling pathway	7	0.0167
hsa03430:Mismatch repair	3	0.0478
